# Adaptative response to changes in pyruvate metabolism on the epigenetic landscapes and transcriptomics of bovine embryos

**DOI:** 10.1038/s41598-023-38686-6

**Published:** 2023-07-17

**Authors:** Aldcejam Martins da Fonseca Junior, Jessica Ispada, Erika Cristina dos Santos, Camila Bruna de Lima, João Vitor Alcantara da Silva, Erika Paulson, Daniel Estanislao Goszczynski, Marcelo Demarchi Goissis, Pablo Juan Ross, Marcella Pecora Milazzotto

**Affiliations:** 1 Federal University of ABC – Center for Natural and Human Sciences, Av. Dos Estados, 5001, Bairro Santa Terezinha, Bloco A, Lab 504-3, Santo André, SP CEP: 09210-580 Brazil; 2 Université Laval, Quebec, Canada; 3 Faculty of Veterinary Medicine and Animal Science, University of Sao Paulo, Sao Paulo, Brazil; 4 Department of Animal Science, University of California, UC – Davis, Davis, USA

**Keywords:** Reprogramming, Epigenetics, Metabolomics

## Abstract

The epigenetic reprogramming that occurs during the earliest stages of embryonic development has been described as crucial for the initial events of cell specification and differentiation. Recently, the metabolic status of the embryo has gained attention as one of the main factors coordinating epigenetic events. In this work, we investigate the link between pyruvate metabolism and epigenetic regulation by culturing bovine embryos from day 5 in the presence of dichloroacetate (DCA), a pyruvate analog that increases the pyruvate to acetyl-CoA conversion, and iodoacetate (IA), which inhibits the glyceraldehyde-3-phosphate dehydrogenase (GAPDH), leading to glycolysis inhibition. After 8 h of incubation, both DCA and IA-derived embryos presented higher mitochondrial membrane potential. Nevertheless, in both cases, lower levels of acetyl-CoA, ATP-citrate lyase and mitochondrial membrane potential were found in blastocysts, suggesting an adaptative metabolic response, especially in the DCA group. The metabolic alteration found in blastocysts led to changes in the global pattern of H3K9 and H3K27 acetylation and H3K27 trimethylation. Transcriptome analysis revealed that such alterations resulted in molecular differences mainly associated to metabolic processes, establishment of epigenetic marks, control of gene expression and cell cycle. The latter was further confirmed by the alteration of total cell number and cell differentiation in both groups when compared to the control. These results corroborate previous evidence of the relationship between the energy metabolism and the epigenetic reprogramming in preimplantation bovine embryos, reinforcing that the culture system is decisive for precise epigenetic reprogramming, with consequences for the molecular control and differentiation of cells.

## Introduction

Early embryogenesis is crucial for development of a healthy offspring as, during this period, embryos undergo remarkable changes in metabolic and epigenetic profiles which may alter embryo fate in the long term^1^. In bovine embryos, prior to morulae compaction, energy metabolism is mainly based on oxidative phosphorylation (even in a scenario of immature mitochondria) due to the low energy requirement. Glycolysis gradually takes place after the major genome activation which coincides with the higher energy demand for cell proliferation, blastocele formation and blastocyst expansion^2^.

The end-product of glycolysis is pyruvate, a key metabolite involved in energy production and biosynthesis in mammalian preimplantation embryos. Its fate during preimplantation development has been extensively studied in several species, including bovine embryos. Pyruvate is primarily utilized as a substrate for oxidative phosphorylation and is converted to acetyl-CoA via the pyruvate dehydrogenase complex (PDC) in the mitochondria. On the other hand, glycolysis yields two molecules of ATP/molecule of glucose, whereas oxidative phosphorylation yields > 30 molecules of ATP/molecule of glucose. The choice between these two pathways has important consequences for the energy metabolism of the embryo^3,4^.

Modulation of pyruvate levels within in vitro culture media has been shown to impact bovine in vitro production. Several studies have shown that supplementation of in vitro culture media with pyruvate can improve embryo development and quality, potentially due to increased energy production and reduced oxidative stress. Conversely, depletion of pyruvate from the culture media has been associated with decreased embryo development and quality. However, excessive levels of pyruvate can be detrimental to embryonic development, potentially due to increased cytoplasmic lactate levels and decreased PDC activity^5–7^.

Although embryonic cells are known by their ability to adapt to nutrient availability, maintaining the function of essential energy-related pathways to survive, these cells encounter other metabolic requirements beyond ATP production. In embryonic stem cells, intermediate metabolites of the TCA cycle, such as alpha-ketoglutarate and succinate, guide chromatin modifications as H3K27me3 and DNA methylation, regulating cell pluripotency^8^. The same way, modulation of the glycolytic pathway and consequently higher availability of acetyl-CoA (the major acetyl donor for histone acetylation) is sufficient to alter the pattern of histone acetylation, mantaining pluripotency, while its loss leads to differentiation^9^. Beyond the generation of acetyl-CoA directly from pyruvate, it can also be converted from citrate through the ATP-citrate lyase, in both mitochondria and nucleus, leading to changes in histone acetylation^10^. More recently, our group showed that this relationship between metabolism and the epigenetic profile is not exclusive of somatic and stem cells, but indeed is involved in the control of the unique epigenetic reprogramming of the pre-implantation bovine embryo^11,12^.

Epigenetic reprogramming in early bovine embryos includes both DNA methylation and post-translational histone modifications (PTMs), which, in general, occurs to erase highly repressive marks derived from gametes, given raise to totipotent cells, followed by the insertion of more specific marks to allow the differentiation of pluripotent blastomeres of the inner cell mass (ICM) and trophectoderm (TE). The dynamics involved in histone acetylation and methylation are among the main mechanisms for epigenetic reprogramming of gametes after fertilization to establish a totipotent state for normal development^12,13^. Acetylations are the most abundant post-translational modifications in histones and are usually related to the reorganization of chromatin to its active form by changing its charge^14,15^, and the degree of such a modification correlates with the level of transcription^16^. Differently from acetylations, histone methylations are not sufficient to alter their charge. In this case, the insertion or removal of methyl groups is reflected on the ability to recruit effector proteins to specific chromatin sites^17^. In both cases, histone post-translational modifications may affect the recruitment of transcription factors or alter chromatin structure, modulating gene expression^18^.

Early embryos are highly sensitive to environmental changes during the period they undergo epigenetic reprogramming. Thus, supported by the metaboloepigenetics concept^19^, in this study we explore the consequences of modulating the pyruvate metabolism during the pre-implantation development to the pattern of global epigenetic marks and their consequences to the molecular status of blastocysts. To investigate this, the metabolic profile of blastocysts was examined following exposure to DCA and IA. Furthermore, global profiles for histone acetylation and methylation were assessed, together with RNAseq and cell differentiation analysis to verify the consequences to the molecular control of embryonic cells.

## Materials and methods

All experimental procedures were performed in accordance with the Animal Research Committee guidelines of the Federal University of ABC. All products were obtained from Sigma Aldrich, unless otherwise stated.

### Embryo production (IVP)

Bovine ovaries were obtained from a commercial slaughterhouse and transported in warm sterile saline [0.9% (w/v) NaCl] for about 3 h. Cumulus-oocyte complexes (COCs) were collected by aspiration of follicles with diameter between 4–8 mm and transferred in groups of 25–30 to 90 μL droplets of maturation medium (TCM-199 bicarbonate supplemented with 10% of FBS, 0.5 μg/mL FSH [Folltropin-V, Bioniche, Belleville, Canada], 100 IU/mL hCG [Chorulon, Merck Animal Health, Boxmeer, The Netherlands]) under mineral oil. COCs were maintained in an incubator at 38.5 °C, 5% CO_2_ in air and high humidity for 24 h.

For in vitro fertilization, frozen semen straws from 2 bulls (donated by a commercial semen processing unit) were thawed in a water bath for 30 s at 37 °C and centrifuged on a discontinuous Percoll gradient (45–90%). The pellet was washed in IVF medium, and the sperm concentration was adjusted to 1 × 106 sperm/mL^20^. Matured COCs were inseminated with 4 μL of semen and incubated at the same conditions of in vitro maturation for 18 h. By the end of fertilization, presumptive zygotes were mechanically denuded by pipette and transferred in groups of 25–30 to 90 µL droplets of culture medium for bovine embryos (IVF Bioscience©, Falmouth, United Kingdon) and placed in an incubator at 38.5 °C, 5% O_2_, 5% CO_2_ in nitrogen and high humidity for 120 h (5 days). After this period, groups of 20 to 30 morula and compact morula were randomly allocated into one of the three experimental groups: Control group—SOFaa medium supplemented with 4% BSA from D5 until D7; DCA group—SOFaa medium supplemented with 4% BSA and 2 mM dichloroacetic acid (DCA) (2156–56-1 Sigma-Aldrich, Germany) from D5 until D7 and IA Group—SOFaa medium supplemented with 4% BSA and 2uM of iodoacetic acid (IA) (305–53-3 Sigma-Aldrich, Germany) from D5 until D7. The pH of culture media was assessed to discard any effect caused by changes in this parameter and no significant differences were found.

**Figure 1:**
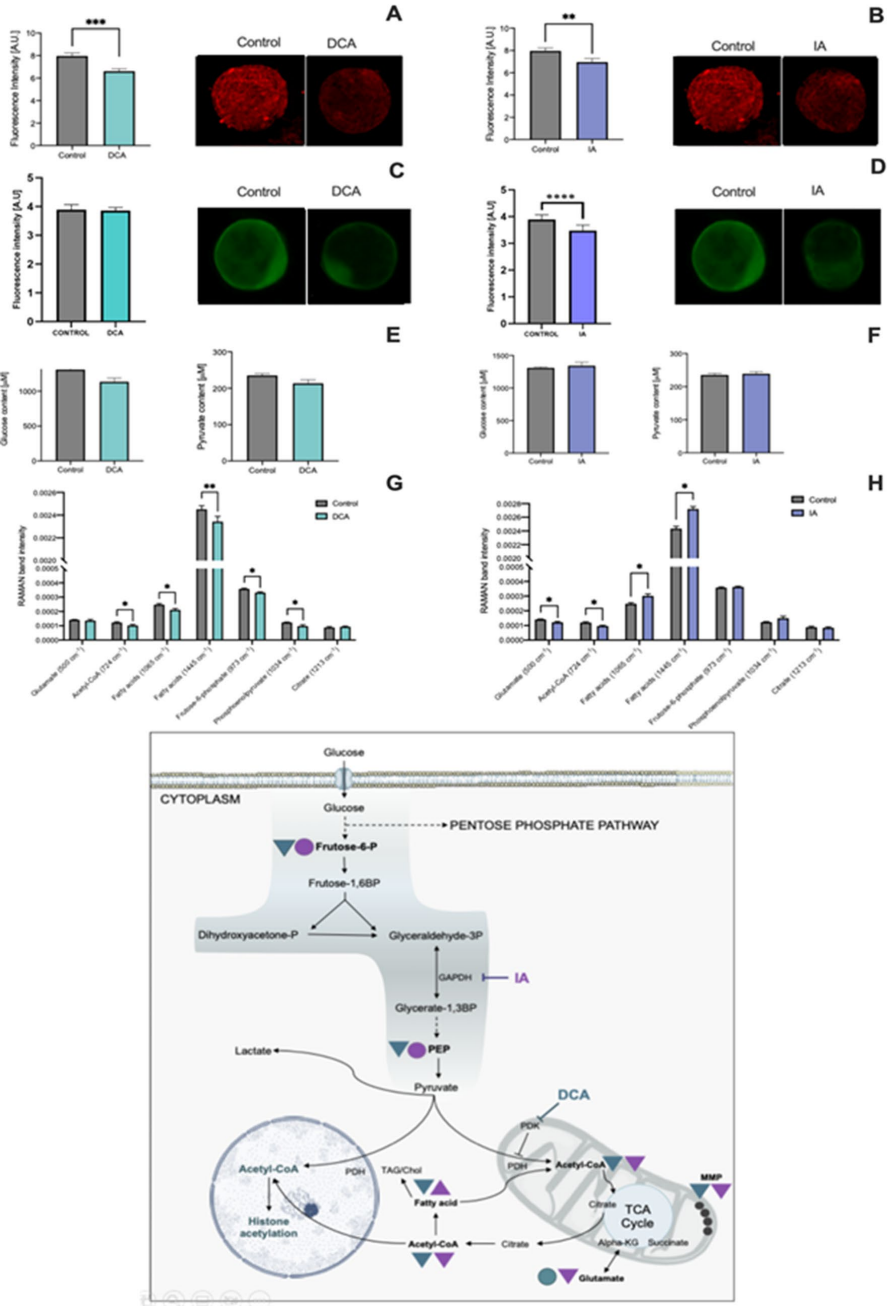
Metabolic analysis of the spent culture media and inner cell masses from the control, DCA and IA groups with the results in graphs and representative images of immunofluorescence of MMP and ACL in whole blastocysts (**A**,**B**) Mitochondrial membrane potential analysis; (**C**,**D**) ATP-citrate lyase; (**E**,**F**) Glucose and pyruvate content in the spent culture media of blastocysts after 12 h incubation; (**G**,**H**) Raman band intensity of inner cell masses; (**I**) Graphical representation of metabolic pathways. In bold, molecules and parameters identified in this study. Triangles represent increase or decrease in DCA (green) and IA (purple) compared to the control group. Circles (green and purple) represent that no statistical difference were found when DCA and IA were compared to control group. Data are represented as mean ± S.E.M. *Represents p < 0.05. **Represents p < 0.01. *** Represents p < 0.001.

DCA is a pyruvate analog that targets the enzyme pyruvate dehydrogenase kinase (PDK), inhibiting the phosphorylation of pyruvate dehydrogenase (PDH), an inhibitor of the conversion of pyruvate to Acetyl-CoA^21^. IA is an inhibitor of glyceraldehyde-3-phosphate dehydrogenase (GAPDH), a glycolytic enzyme that catalyzes the conversion of glyceraldehyde-3-phosphate to 1,3-bisphosphatoglycerate resulting in the production of NADH and glycolysis inhibition^22^ (Fig. 1G). In both cases, we expected that changes in pyruvate metabolism could alter the availability of Acetyl-CoA, altering the availability of acetyl groups for histone acetylation.

### Metabolic assessment

#### Mitochondrial membrane potential

Mitochondrial membrane potential (MMP) was evaluated in D5 and D7 embryos (4 expanded blastocyst/per replicate/per group; n = 4 replicates, each blastocyst was considered an experimental unit) by using the fluorescent probe MitoTracker Red CMXRos (M-7512; Life Technologies, California, USA) according to the protocol specified by the manufacturer. After that, they were washed, mounted on slides, and individually photographed using an epifluorescence microscope (Leica Microsystems DMI6000 B—Excitation/emission 538/617 nm. ImageJ-Fiji (National Institutes of Health, Bethesda, MD, USA) was used to analyze the produced images. For that, the fluorescence channel was measured manually by selecting a specific region of interest (ROI) (predicted ICM area) after background subtraction. Fluorescence was measured to determine its integrated density (IntDen) which corresponds to pixel intensity and considered as the dependent variable of the analysis. Each embryo was considered an experimental unit.

#### Glucose and pyruvate consumption

Quantitative evaluation of glucose and pyruvate in culture media of bovine blastocysts were performed with the aid of a standard curve, to determine the consumption of these metabolites. For that, expanded blastocysts (groups of 5) were transferred to 50 µL droplets of fresh culture medium (SOFaa with addition of the modulators in the case of treatment groups), when they remained for 12 h. After that, the volume (2 droplets/ replicate/ group; total of 3 replicates) was collected and the consumption of glucose and pyruvate were assessed by comercial kits (Amplex™ Red Glucose/Glucose Oxidase Assay Kit (Thermo Fisher, California, EUA) and EnzyChrom Pyruvate Assay (Bioassay systems, California, EUA) based on enzymatic reactions with fluorometric detection according with the manufacturer’s instructions.

As we used a defined culture media for this experiment, consumption was calculated according to the amount of glucose/pyruvate measured in each sample subtracted from the initial quantities of each of those substrates.

#### Metabolomic analysis of inner cell mass by Raman spectroscopy

Expanded blastocysts collected on day 7 of IVC were washed in SOF medium supplemented with 4% BSA and HEPES (SOF-HEPES) and incubated for 1 min in pronase solution (10 mg/mL) to remove the zona pellucida. The blastocysts were then washed 5 times in SOF-HEPES and incubated in 20% anti-bovine serum for one hour at 38.5 °C. After incubation, the embryos were washed 5 times in SOF-HEPES and incubated in 20% guinea pig complement for one hour at 38 °C. The ICM was mechanically isolated by successive pipetting and immediately analyzed.

The Raman spectroscopy setup details and analysis were described elsewhere^23^. Briefly, ten Raman spectra were collected per ICM (total of 3 ICM/ group/ replicate; total of 3 replicates) by using a Triple T64000 Raman Spectrometer (Horiba Jobin–Yvon S.A.S., France) with microanalysis option and CCD detector 1024 × 256—OPEN-3LD/R with quantum response of ∼40%. The excitation laser was 532 nm focused on a spot with 5-mW power. The spectra were acquired by using a plan achromatic 50 × objective glass (0.20 mm∕NA ¼ 0.50) with time of exposure of 20 s, and confocal aperture in 6.5 μm. Data were plotted using the Origin 8.0 software (OriginLab, Northampton, Massachusetts, USA) and pre-processed to remove spikes. The pre-processed data were further analyzed using the Spectrograpy 1.2.15 software to identify the peaks and noise of the spectra with the following parameters: Limit 5% and Prominence 3. Peak attributions were done according to De Gelder, 2007^24^.

### Global epigenetic assessment, total cell number and CDX2 analysis

Immunostainning was performed for the detection of acetylation of H3K9 and H3K27 (H3K9ac, H3K27ac), trimethylation of H3K27 (H3K27me3), ATP citrate lyase (ACL) and CDX2. The description and details of the antibodies used are listed in Supplementary file 1.

Expanded blastocysts were collected on D7 and fixed in 4% paraformaldehyde for 30 min. The embryos were then subjected to permeabilization and blocking before the antibody’s treatment according to Ispada et al., 2020^11^. Incubation with the primary antibodies antihistone H3 (acetyl K9) antibody, anti-acetyl histone H3 (Lys27) antibody, anti-trimethylation histone H3 (Lys 27) antibody, anti-ATP citrate lyase antibody and CDX2 was conducted. After that, the embryos were incubated with the fluorescent secondary antibody IgG H&L (Alexa Fluor® 488) highly cross-adsorbed or IgG H&L (Alexa Fluor® 594) preadsorbed (green—excitation/emission 512–542 nm). Finally, nuclear staining was done with Hoechst 33342 dye (Thermo Fisher, Massachussets, USA), (blue—excitation/emission 350–490 nm). Embryos were placed on a slide containing 20 μL of ProLong® Gold Antifade Mountant (ThermoFisher, Massachussets, USA) and covered with coverslips. For epigenetic marks, a Leica TCS SP8 STED 3X confocal microscopy system was used for image acquisition with section intervals of 10um, by using the Leica® Application Suite software (LAS, v. 3, Leica Microsystems, Germany). An epifluorescence microscope with the z-stack function was used for the acquisition of ACL and CDX2 images (Leica Microsystems DMI6000 B). For CDX2, multiple focal planes were analyzed to visualize and document the distribution patterns and only those nuclei in the focal plane of each image were used for quantification. In the case of ACL, the images were collected in z-stack mode and the fluorescence intensity was analyzed according to Saiz et al. 2016. Briefly, a slope value was used to correct for fluorescence decay along the Z-axis as described previously^25^:

$${\text{Z}}\;{\text{Corrected}}\;{\text{Intensity }} = {\text{ Original}}\;{\text{intensity}} - \left( {{\text{Slope }} \times {\text{ Z stack}}} \right)$$

The slope value was obtained through linear regression considering Z-stack as the independent variable and mean fluorescence intensity as the dependent variable for all measurements. Corrected values were then subtracted by an average of two background values, which were also corrected by Z-axis position.

ImageJ-Fiji (National Institutes of Health, Bethesda, MD, USA) was used to reconstruct and analyze all the images. For that, each fluorescence channel was measured manually by selecting a specific region of interest (ROI) (isolated nucleus) after background subtraction. Fluorescence was measured to determine its integrated density (IntDen) which corresponds to pixel intensity and considered as the dependent variable of the analysis. Each nucleus (minimum of 100, maximum of 360 per antibody per group) was considered an experimental unit. ICM and TE were analyzed separately for epigenetic marks based on nuclear morphology and size (1@@0.1371/journal.pone.0124619) and the morphology of the blastocyst and these data are presented in Supplementary Fig. 2.

### Analysis of the global transcriptomic profile by RNASeq

As at blastocyst stage the metabolic and molecular profile of ICM and TE cells is quite different, we also opted for isolating the ICM for RNASeq analysis^26,27^. RNA isolation, amplification and cDNA synthesis were performed as previously described^28^. Briefly, the RNA for 3 ICM per group per replicate (3 replicates) were extracted using the PicoPure RNA Isolation Kit, including DNAse treatment, following the manufacturer's instructions, but with an RNA elution step modified. The RNAs (7 uL per sample—1.6 ng average) were used for cDNA synthesis and amplification by the Ovation RNA -seq V1 kit (NuGen, San Carlos, CA, USA) following the manufacturer's recommendation. At the end, the samples were again quantified and an average of 1ug of each sample was used to construct the cDNA libraries. Bioanalyzer 2100 (Agilent) was used for quality control.

The samples were sonicated to produce DNA fragments of an average of 300 bp. The libraries were generated by the NEBNext Ultra II DNA Library Prep Kit for Illumina system. Libraries were considered adequate with fragments of an average of 350 bp in size (resulting from fragments and adapters). The samples were sequenced at the UC Davis Genome Sequencing Center by the Illumina Genome Analyzer IIx with 150 bp reads. Quality control of reads and alignment were performed using Genomics CLC Workbench software 4.7 (CLC bio, Aarhus, Denmark). First, the samples were checked for the quality and quantification of the transcripts and the alignment of the reads. Comparisons between the transcripts of each group were made using the EdgeR (Differential Gene Expression Analysis—usegalaxy.org) system and those with p < 0.005, padj < 0.07 and log2FC > 1 or < −1 were considered differently represented between the groups.

DEGs between comparisons were ordered by padj value and the Gene Ensembl IDs of the transcripts for each comparison were subjected to functional annotation by the PANTHER classification system (http://pantherdb.org/). The annotation was conducted with Bos taurus background, and the grouping was performed based on "biological processes". After the annotation, the most enriched pathways were identified.

### Statistical analysis

Statistical analyses were performed comparing each treatment (DCA or IA) with the control group in a Prisma 5 environment (GraphPad Software Inc., USA) for all data, except for RNASeq. The results are presented as graphs with their respective units or in Arbitrary Units (U.A.). All data were initially submitted to an outlier detection test (Grubbs test with ROUT Q = 1%) and the Shapiro–Wilk normality test (alpha = 5%). H3K9ac, H3K27ac were non-parametric and were transformed (log2(Y)) and further analyzed by t-Student test. H3K27me3, ACL, MMP, glucose and pyruvate consumption and Raman peaks intensity were analyzed by Mann–Whitney test. Groups with P ≤ 0.05 in relation to the control were considered different.

### Animal studies ethics statement

This study uses bovine ovaries obtained from slaughterhouse. The Center for Natural and Human Sciences (CCNH) of the Federal University of ABC or the Research Foundation of Sao Paulo did not require the study to be reviewed or approved by an ethics committee because the source is a by-product of the livestock industry.

## Results

### Metabolic interference alters mitochondrial function and acetyl-CoA availability

To understand the metabolic remodeling imposed by changes in pyruvate metabolism during preimplantation embryonic development, we assessed four parameters as follows: the consumption of glucose and pyruvate from the culture media, the mitochondrial membrane potential, the quantification of ATP-citrate lyase immunofluorescence and the metabolomics by RAMAN spectroscopy of the ICM (Fig. 1). The latter was chosen as it allows the characterization of a single ICM, diminishing the variability of results (easing to spot an outlier) as well as focusing on the compartment more associated with the aimed changes.

Bovine blastocysts from DCA group consumed glucose and pyruvate from culture media similarly to control embryos (last 12 h of in vitro culture) (Fig. 1E) but presented lower levels of fructose-6-P (Fig. 1G) in the ICM, what could suggest a premature turn, making a higher influx to the pentose phosphate pathway, rather than the glycolytic pathway. This deviation, also reinforced by lower levels of PEP and an unexpected decrease in acetyl-CoA levels, seems to give us a clue of a possible adaptive response to DCA which could lead, at first, to greater levels of these metabolites, disturbing energy metabolism. This hypothesis was further corroborated by the analysis of the mitochondrial membrane potential after 8 h of incubation in DCA, still at the morula stage, which led to an increase in this parameter (Supplementary Fig. 1A and 1B). The lower levels of acetyl-CoA in blastocysts were followed by lower levels MMP (Fig. 1A,C).

In the case of IA-derived blastocysts, as expected, the pharmacological impairment of the glycolytic pathway led to a decrease in acetyl-CoA (Fig. 1H). This lower influx of acetyl-CoA to the TCA cycle was followed by lower MMP (Fig. 1B) and lower ACL levels (Fig. 1D). An increase in RAMAN band intensity related to fatty acids as myristic and palmitic acids may suggest an attempt of cells to maintain the energy production by mobilizing fatty acids through beta-oxidation (Fig. 1H).

### Perturbations aiming pyruvate metabolism alter epigenetic histone reprogramming

**Figure 2:**
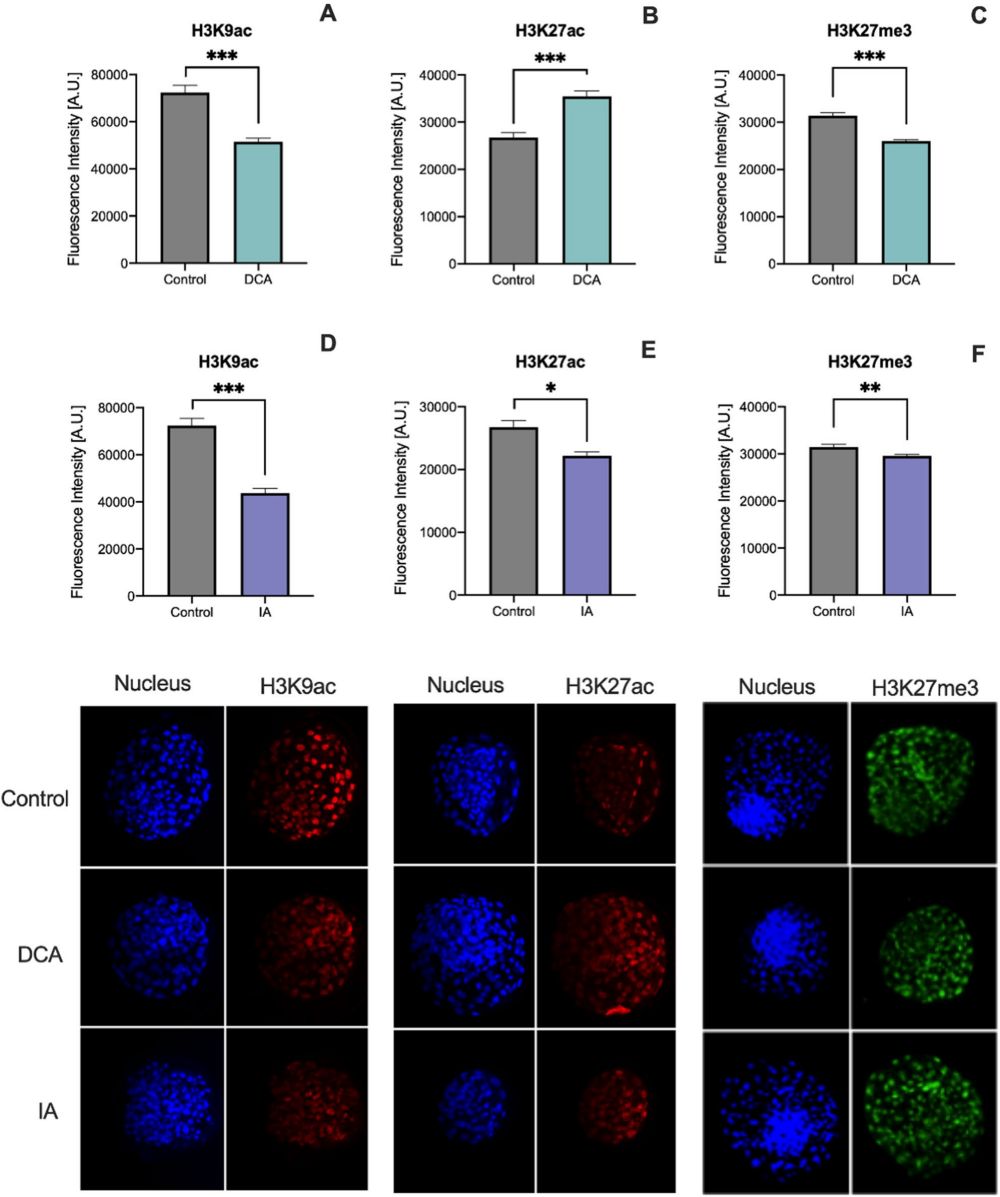
Fluorescence intensity levels and representative images (original magnification 400×) of H3K9ac (**A**,**D**), H3K27ac (B and E) and H3K27me3 (**C**,**F**) in blastocysts from the DCA (**A**,**B**,**C**) and IA (**D**,**E**,**F**) groups compared to control group. Nuclei were stained with Hoescht 33,342 and immunostained with antibodies against H3K9ac, H3K27ac or H3K27me3. Data are represented as mean ± S.E.M. *Represents p < 0.05. **Represents p < 0.01. *** Represents p < 0.001.

From the moment of the major EGA until blastocyst stage, bovine embryos are highly dynamic in terms of epigenetic reprogramming both in histones and DNA. More specifically, it is expected a decrease in H3K9ac^12^ and an increase in H3K27ac, while H3K27me3 is expected to maintain the low level achieved at 8–16 cell stage compared to 2–4 cells^29^. Histone methylation and acetylation, beyond the activity of specific epigenetic-related enzymes, depend on the availability of metabolic intermediates, as acetyl groups, S-adenosylmethionine, succinate and others, which led us to investigate the consequences of changes in pyruvate metabolism on the establishment of these marks (Fig. 2). Lower levels of H3K9ac were found in both DCA and IA-derived blastocysts (Fig. 2A,D). When enriched ICM region was analyzed separately, the same pattern was observed, suggesting that the lower acetyl-CoA levels found in these groups had a decisive impact on H3K9ac establishment (Supplementary Fig. 2A,B).

H3K27ac and H3K27me3 are competitive marks, and it was already described in embryonic stem cells that the loss of H3K27me3 results in an increase in H3K27ac, followed by transcriptional activation^30^. In our study, embryos cultured in DCA gave rise to blastocysts with higher levels of H3K27ac (Fig. 2B) and lower levels of H3K27me3 (Fig. 2C), indicative of an overall more permissive chromatin structure. Enriched ICM region analysis revealed the same pattern for H3K27ac, but interestingly no changes found in H3K27me3 when compared to control (Supplementary Fig. 2C and 2E). For blastocysts from the IA group, lower levels of H3K27ac and H3K27me3 were found (Fig. 2E,F), however, when enriched ICM regions were analyzed separately, an inverted pattern was observed, with lower levels of H3K27ac but higher levels of H3K27me3, indicating the competitive profile previously described (Supplementary Fig. 2D,F). These data point to pyruvate metabolism as a distinguished apparatus capable of impacting the regulation of histone post-translational modifications in early embryos.

### Epigenetic changes induced by metabolic adaptation impact the global transcriptomic profile of bovine embryos

**Figure 3:**
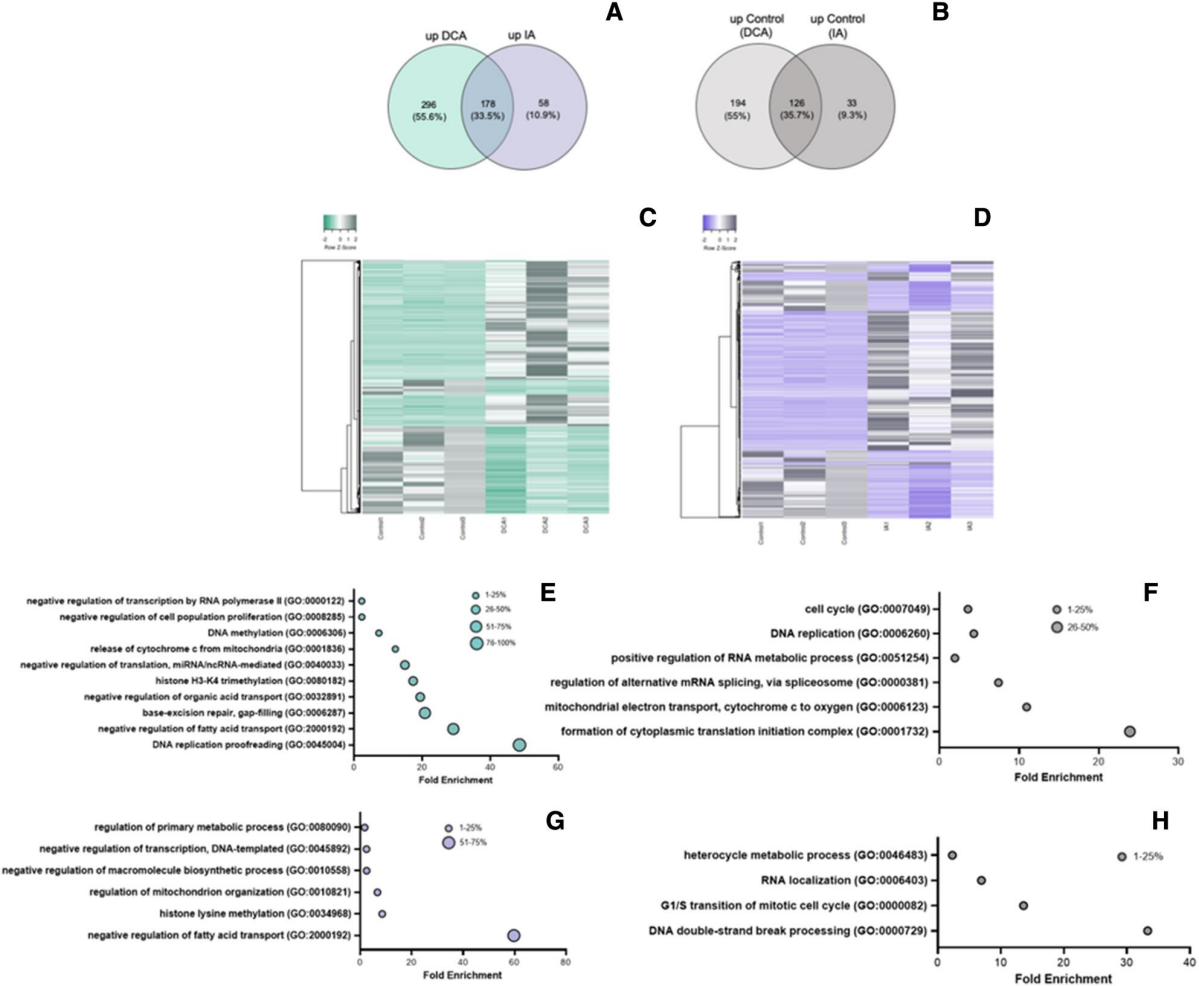
Molecular evidence for DCA, IA and control group. (**A**) Venn diagram of exclusive upregulated transcripts in DCA group (green), exclusive upregulated transcripts in IA group (purple) and common upregulated transcripts (merged) when treatments were compared to control. (**B**) Venn diagram of exclusive upregulated transcripts in control group when compared to DCA group (light gray), exclusive upregulated transcripts in control group when compared to IA group (dark gray) and common upregulated transcripts (merged) in both comparisons. (**C**) Heat map of DEGs and linkage analysis of groups for the comparison Control vs. DCA. (**D**) Heat map of DEGs and linkage analysis of groups for the comparison Control vs. IA. The most affected upregulated biological processes are indicated in (**E**) for DCA, (G) for IA, (**F**) for the control group compared to DCA and (G) for the control group compared to IA. The circle’s size indicates the percentage of altered transcripts in relation to the number of transcripts belonging to the pathway (Images generated using Heatmapper: //www.heatmapper.ca/ and Veinny 2.1.0: //bioinfogp.cnb.csic.es/tools/venny/).

To address the transcriptional regulation related to epigenomic changes during preimplantation development, we explored the transcript profiles of isolated ICM by RNASeq analysis. The transcripts upregulated in both DCA and IA were 178, while the concurrent in their respective control groups were 126 (Fig. 3A–D). A total of 794 transcripts were differentially represented between control and DCA groups. From those, 474 were upregulated in DCA and 320 in control. The analysis of the biological processes upregulated in the DCA group revealed important metabolic pathways as “lipids/fatty acids transport, localization and metabolism” and “regulation of organic acid transport”, “DNA replication and repair”, “inhibition of translation mediated by ncRNA and miRNA”, “Inhibition of transcription”, “programmed cell death” and “DNA/histone methylation” (Fig. 3E). Upregulated biological processes in the control group were related to “transcription and RNA processing”, “translation, protein folding and localization”, “mitochondrial electron transport” and “cell cycle” (Fig. 3F and supplementary table 1).

Comparison between control and IA-derived ICMs revealed 395 differentially represented transcripts, 236 upregulated in IA and 159 in control group. As for the DCA group, “regulation of fatty acid/lipid transport” and “histone methylation” were among the most affected biological processes upregulated by the treatment. “Biosynthetic process”, “regulation of transcription and RNA metabolism” and “regulation of primary metabolism” were also affected by IA (Fig. 3G). Biological processes related to cell functioning and proliferation were upregulated in the control group, as “DNA and chromatin organization and repair”, “cell cycle progression”, “RNA processing” and “metabolism” (Fig. 3H and supplementary table 2).

### Metabolic adaptation to changes in pyruvate metabolism supports embryo development but impairs the pluripotent network in blastocysts

**Figure 4:**
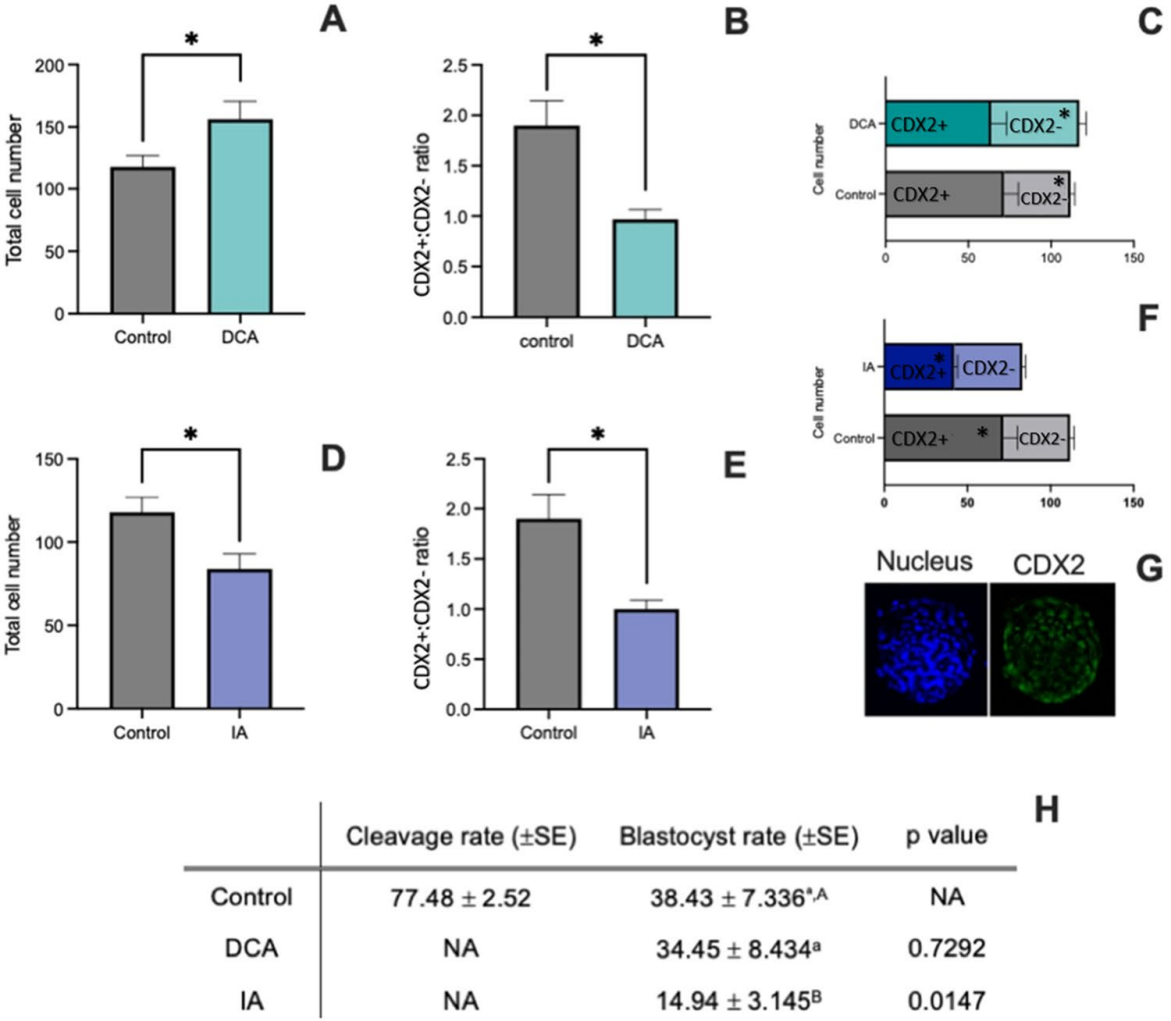
Total cell number of blastocysts from DCA (**A**) and IA (**D**) groups when compared to control group. In (**B**) and (**E**) are presented the CDX2+ :CDX2− ratio assessed by the number of Hoescht 33342 and CDX2 positive cells from DCA and IA blastocysts when compared to control. In (**C**) each column represents the total number of cells and the number of CDX2+ and CDX2− cells separately for DCA and control group. *Represents p < 0.05 for the comparison between the groups for the same cell type (i.e. CDX2 + DCA vs. CDX2 + control). In (**F**) each column represents the total number of cells and the number of CDX2 + and CDX2- cells separately for IA and control group. *Represents p < 0.05 for the comparison between the groups for the same cell type (i.e. CDX2 + DCA vs. CDX2 + control). (**G**) representative images (original magnification 400 ×) of Hoescht 33,342 (Nucleus) and CDX2 staining. (**H**) Cleavage and blastocyst rates. Cleavage is presented as a single value since the supplementation was performed only in D5 of culture. For blastocyst rates, lowercase letter represent the comparison between control and DCA and uppercase letters the comparison between control and IA. Different letter represents p < 0.05.

Early developing embryos have the ability to alter their preferred substrate for oxidation in response to the maternal/in vitro environment, nevertheless, this adaptation may have a cost. To address the capability of embryos to properly develop after metabolic adaptation, we verified the blastocyst rate, total cell number and CDX2 positive cells, aiming to indicate a possible relationship encompassing metabolism, epigenetic changes and consequences for cell proliferation and differentiation (Fig. 4). Both DCA and IA-derived embryos were able to develop until the blastocyst stage, despite this ability was inferior in the IA group (Fig. 4H). The total cell number was higher in DCA blastocysts and lower in IA (Fig. 4A,D), corroborating the “cell cycle” as one of the affected biological processes identified by RNASeq. Both DCA and IA blastocysts presented a lower CDX2 + :CDX2- ratio (Fig. 4B,E,C,F), possibly indicating differences in TE:ICM ratio in treated groups when compared to the control. This data corroborates previous work which demonstrated that bovine TE cells present a higher glycolytic activity when compared to ICM cells^28^. In the present work, disturbing the pyruvate metabolism may have led to a decrease of the glycolytic dependent lineage as a consequence.

## Discussion

In the present work we demonstrate that, under stress conditions caused by the modulation of pyruvate metabolism, embryos find distinct metabolic pathways to maintain the energy homeostasis and development. The consequences of that impinge on epigenome reprogramming, disturbing the molecular pattern and cell differentiation, reinforcing the need of considering the dynamic of embryonic metabolism to further improve production systems to maximize the embryo viability and progeny health. The approach we utilized for integration of data generated from culture medium, whole bovine blastocysts, and isolated inner cell mass allows us to work on the challenge of foreseeing a more complete picture of the system and validate findings across different methods. Despite the challenges, we have worked on careful experimental design, data presentation and statistical analysis to look deeply into the complex processes that underlie embryo metabolism, epigenetic reprogramming and the intrinsic molecular mechanisms in this relationship.

During the mammalian preimplantation embryogenesis, fundamental processes to assure the proper development are dependent on accurate metabolic instructions. More specifically, after morula compaction, blastocysts present higher levels of metabolites related to the mitochondrial tricarboxylic acid cycle and a more oxidative state, compatible with the resumption of mitochondrial biogenesis^31,32^. The pyruvate metabolism occupies a central position in the blastocyst energy generation as it connects two of the most important pathways in this stage, the glycolytic pathway, and the TCA cycle. Also, TCA cycle was recently described as an important regulator of the embryonic epigenome^33^. In this work, we address the consequences of manipulating the pyruvate metabolism during the embryo development and propose a comprehensive profiling of embryonic metabolic, epigenetic, and molecular adaptation to these alterations.

DCA is known as a stimulator of the oxidative metabolism by inhibiting the kinase responsible for the phosphorylation and inactivation of the pyruvate dehydrogenase complex^34^, augmenting the pyruvate to acetyl-CoA conversion. This acetyl-CoA may enter the TCA cycle, increasing the generation of intermediates for oxidative phosphorylation as well as other indispensable metabolites, important for several cellular functions, as citrate, which can also be converted back to acetyl-CoA by ACL^10^. Acetyl CoA is also the substrate for histone acetyltransferases, regulating gene expression. In this work, the supplementation with DCA for 48 h resulted in diminished levels of acetyl-CoA in ICM of blastocysts, as well as its glycolytic precursors as fructose 6-phosphate and phosphoenolpyruvate.

This unexpected reduction may occur to reduce an initial mitochondrial overload occurring at the time of supplementation, as shown in Supplementary Fig. 1. In mouse embryos, the supplementation with 1 mM DCA during embryo culture resulted in blastocysts with increased mitochondrial membrane potential, reactive oxygen species, pyruvate oxidation and adenosine triphosphate (ATP) content^35^. The day 5 of development is a critical time for bovine embryonic survival as the metabolic shift occurs and the embryos commence to metabolize glucose more efficiently via the glycolytic pathway. Excess of glucose in the culture medium at this stage, for example, led to a decrease in mitochondrial membrane potential, followed by higher levels of stress markers and diminished embryonic development^36^. It is noteworthy that transcripts for SLC2A8, one of the important glucose transporters of this phase, are also decreased and 6-phosphogluconolactonase (PGLS), the second PPP enzyme which converts 6-phospho-delta-gluconolactone to 6-phosphogluconate (6PG) is upregulated, suggesting that these changes in transcripts may be directly related to the biochemical activity in charge of aiming at the preference of the glucose-derived intermediates to this pathway. Thus, metabolic control in an attempt to bypass this mitochondrial overload may be leading to a reduction in the synthesis of glycolytic intermediates in ICM.

Oppositely to the decrease of these intermediates and transporter, the levels of transcripts of PFK, ALDOA and ENO, all belonging to the glycolytic pathway, were found to be increased in the DCA group, suggesting a possible post-transcriptional (supported by the enrichment of the mRNA degradation pathway) or even a post-translational control of these enzymes. The control of glycolytic pathway activity by post-transcriptional processes is well described, especially in tumor cells^37^. In these cells, ZFP36 (also known as TPP), which is upregulated in the DCA group, is responsible for post-transcriptionally downregulate PFKFB3 and its overexpression contributes to suppression of glycolysis^38^.

Another route for the generation of acetyl-CoA is the oxidation of fatty acids through the beta-oxidation process, which can occur in both mitochondria and peroxisomes. In ICM of DCA-derived blastocysts we observed a decrease in the amount of fatty acids coupled with the upregulation of pathways related to lipid metabolism, including transcripts involved in fatty acid esterification and cholesterol synthesis, transport and storage, such as NR1H2, SREBP, APOA1 and DISP3, as well as for the storage of lipids such as PPARG^39^. Taken together, these results can indicate lipid accumulation rather than oxidation. It is important to emphasize that the inhibitory effect of medium-chain and short-chain fatty acid oxidation caused by DCA has been previously described in rat heart mitochondria^40^.

This metabolic adaptation to control a possible exacerbated initial increase in mitochondrial activity by downregulating the glycolytic pathway and beta-oxidation resulted in a decrease in mitochondrial membrane potential, but still sufficient to maintain the development until blastocyst stage. In addition, our data suggest that mitochondrial activity must be supported by other fuels such as amino acids. In fact, several transcripts of amino acid transporters were found to be upregulated in the DCA group such as SLC43A1, SLC6A20, SLC7A1, SLC7A4, SLC7A5, SLC25A11 and SLC25A22. SLC25A22 transports glutamate into the mitochondria, where it can be converted to alpha-ketoglutarate and used in the TCA cycle. This alternative method has already been described in tumor cells as a pathway to maintain cell viability, allowing survival during impaired mitochondrial pyruvate transport^41^. Despite that, ICMs of DCA-derived blastocysts had upregulation of apoptosis related pathways, especially those involving mitochondria, as “apoptotic mitochondrial changes” and “release of cytochrome c from mitochondria”.

SLC25A11, an oxoglutarate:malate antiporter, allows the malate-aspartate shuttle (MAS) regulating metabolic activity. The MAS was described as a key regulator of embryo metabolism, maintaining its viability even in the absence of pyruvate by transferring reducing equivalents derived from glycolysis and lactate metabolism from cytosol into mitochondria^42^. This activity is essential especially for survival in metabolic stress conditions in which mitochondrial NADH is required for ATP production^43^. Also, when MAS is inhibited, the rate of glycolysis and lactate production is elevated, showing a direct relationship between these two metabolic pathways^44^. In the case of this study, MAS could be an alternative pathway to preserve oxidative phosphorylation, escaping from acetyl CoA synthesis.

An alternate pathway to avoid the pathological excess of acetyl CoA could be the increase in citrate synthesis, a six-carbon molecule with three carboxyl groups. This citrate can either remain in the TCA cycle or be exported from the mitochondria, being directed to lipid synthesis (reviewed by^45^). This latter is suggested in the DCA group by the upregulation of transcripts involved in lipid synthesis and storage pathways, coupled with the high levels of SLC25A1, responsible for the export of citrate from the mitochondria, even though, no difference in citrate was found by Raman spectroscopy.

In addition to the metabolic effects observed, DCA supplementation was also expected to have a consequence for the molecular control of the cell, since changes in the availability of acetyl-CoA can have the direct effect altering the pattern of histone acetylation, leading to disturbances in molecular control. In fact, the decrease in acetyl-CoA walked together with a decrease in H3K9ac levels, increase in H3K27ac and a decrease in H3K27me3, although the latter was not evidenced analyzing the enriched ICM region separately. The decrease in H3K9ac, albeit in accordance with the lower acetyl CoA in the enriched ICM region of blastocysts, was not expected as previous results from our group showed that at the dose of 2 mM DCA was capable of increasing this mark in blastocysts. However, it is important to mention that the culture media used in the present work was different in regard to glucose, pyruvate, lactate and amino acids when compared to the previous one, which may lead to different metabolic adaptation^12,46^. Despite that, there seems to be a contribution of metabolism to the epigenome reprogramming of embryonic cells. Interestingly, the acetylation of the two lysines analyzed in this study had opposite patterns. This fact may be related to specific biological functions of each of these modifications. While H3K9ac is associated with active promoters and is considered a trademark of active transcription^47^, H3K27ac is mainly located in the enhancer, distinguishing those active from inactive/poisoned ones^48^.

Regarding H3K9ac, its reduction is associated with cell differentiation, and is even capable of predicting pluripotency in embryonic stem cells^49,50^. Although this fact contrasts with the greater number of ICM cells found in embryos from the DCA group, when we assume that in the short term there was an increase in the amount of acetyl CoA and possibly an increase in the histone acetylation profile, this could have delayed or inhibited differentiation to TE cells. Corroborating this hypothesis, lower levels of transcripts for ROCK1 and ROCK2 were found in enriched ICM regions of DCA group, which could have led to CDX2 inhibition, as already reported in mouse embryos^51^. Importantly, histone acetylation is a more dynamic epigenetic modification in cells^52^.

In spite of the increase in H3K27ac, this modification when located in enhancer regions seems to not have a decisive impact on embryo’s cells transcriptome, as reported for embryonic stem cells^53^. Despite that, this mark may also be present at transcription start sites of actively transcribed genes marked by trimethylated H3K4 (H3K4me3) (reviewed by^54^). Interestingly, higher levels of transcripts involved in H3K4me3 were present in the ICMs of these embryos, indicating a highly permissive chromatin scenario. As both acetylation marks assessed in this study presented opposite profiles and no differences were observed in transcripts related to the insertion and removal of these specific marks, more studies are necessary to elucidate the exact region of these modifications, other associated epigenetic marks as well as additional layers of control other than the availability of acetyl donor groups and enzyme-related transcripts.

Iodoacetate, an inhibitor of the glycolytic enzyme GAPDH, was also used in this study to verify how the impairment of pyruvate metabolism could interfere in epigenetic and molecular reprogramming. As previously reported by our group, the use of 2uM iodoacetate from day 5 of culture reduced the levels of H3K9ac^12^. In the present work, this reduction was accompanied by lower levels of H3K27ac and H3K27me3 in whole blastocysts. When enriched ICM regions were analyzed separately, H3K9ac and H3K27ac were lower and H3K27me3 were higher in this group. As expected, lower levels of histone acetylation promoted a transcriptionally repressive chromatin state corroborated by the enrichment of pathways related to the “negative regulation of transcription and RNA processing” in IA group and “RNA processing and gene expression” in ICMs of control group^55^.

Lower levels of global histone acetylation may be the consequence of the lower acetyl CoA observed in this group, which was accompanied by lower MMP, suggesting an overall reduction in mitochondrial activity. It is important to emphasize that this impairment in pyruvate metabolism, probably led to the deviation of other metabolites in an attempt to maintain the mitochondrial metabolism. One indicator of that is the higher amount of fatty acids found in this group, together with the enrichment of the “regulation of fatty acid transport “ pathways due to higher levels of transcripts related to fatty acid metabolism as AKT1, AKT2, FIS1 and FASN. Indeed, in endothelial cells submitted to a hypoglycemic environment, there is an increase in ROS generation through activation of fatty acid oxidation^56^. In placental cells, high glucose levels reduced mitochondrial fatty acid oxidation leading to accumulation of triglycerides^57^. In oocytes, the supplementation of culture media with a mixture of fatty acids caused a reduction in glucose uptake, indicating that higher fatty acid oxidation is related to inhibition of carbohydrate metabolism^58^.

Another indicator of the effect on mitochondrial function is the low level of PHGDH transcript in IA group. PHGDH is an enzyme involved in the early steps of L-serine synthesis, catalyzing the oxidation of 3-phospho-D-glycerate (an intermediate of the glycolytic pathway) to 3-phosphonooxypyruvate^59^. In the case of the IA group, this could represent an attempt to deviate more intermediates to the glycolytic pathway instead of L-serine synthesis. PHGDH is also required to maintain nucleotide synthesis by supporting central carbon metabolism^60^. In this sense, a consequence of that is enrichment of pathways as “negative regulation of RNA processes and gene expression” and an enrichment of “RNA processing” and “nitrogen compound metabolic processes” an “cell cycle related pathways” in ICMs of control blastocysts, which corroborates the lower CDX2 + :CDX2- ratio found in this group.

Taken together, our results indicate the remarkable plasticity of mammalian embryos, highlighted by the ability to gather distinct metabolites and pathways aiming at reaching energy homeostasis and development. These events, nevertheless, may lead to an epigenetic and molecular reorganization with consequences to cell fate specification and embryo viability. It is intelligible that changes induced by metaboloepigenetics approaches impact embryonic development, and this work contributes to shed light on the understanding of the network involved in embryo reprogramming and how the adequate modulation of environmental stimuli may produce a better-quality blastocyst.

## Supplementary Information

Supplementary Information.

## Data Availability

The datasets generated during and/or analysed during the current study are available from the corresponding author on reasonable request. Some data generated or analysed during this study are included in this published article (and its Supplementary Information files).
