# Supplementary material for: Adaptative response to changes in pyruvate metabolism on the epigenetic landscapes and transcriptomics of bovine embryos
Source: Sci Rep. 2023 Jul 17;13:11504. doi: 10.1038/s41598-023-38686-6 (PMC10352246; doi:10.1038/s41598-023-38686-6)
Supplement: Supplementary file 1 — Supplementary Information. [file 41598_2023_38686_MOESM1_ESM.docx]

| Description | Source | Cat Reference | Incubation and concentration |
| --- | --- | --- | --- |
| Anti-histone H3 (acetyl K9) | Abcam, UK. | ab12179 | Overnight (4ºc)– 1:1000 |
| Anti-acetyl histone H3 (Lys27) | Millipore,USA | 05-1334 | Overnight (4ºc)– 1:1000 |
| Anti-trimethylation histone H3 (Lys 27) | Abcam, UK. | ab6002 | Overnight (4ºc)– 1:1000 |
| Anti-ATP citrate lyase | Abcam, UK. | ab157098 | Overnight (4ºc)– 1:1000 |
| Secondary antibody IgG H&L Alexa Fluor® 568 | Invitrogen, USA | A-11031 | 1 hour (room temperature) – 1:400 |
| Secondary antibody IgG H&L Alexa Fluor® 488 | Invitrogen, USA | A-21206 | 1 hour (room temperature) – 1:400 |
| CDX2 primary antibody | Santa Cruz Biotechnology, USA | sc166830 | Overnight (4ºc) – 1:50 |

**Supplementary table 1 –** List with description of antibodies used with their respective

source and CAT number.


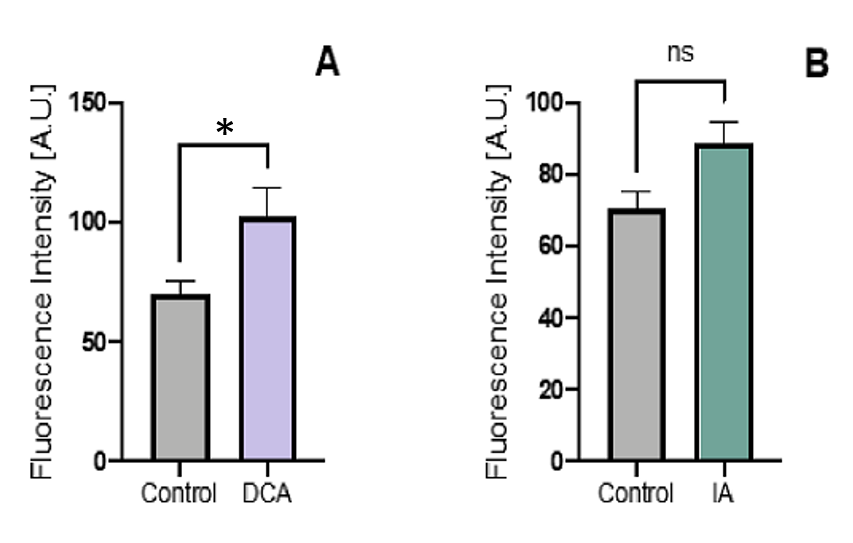


**Supplementary Figure 1** - (A and B) Mitochondrial membrane potential analysis of embryos after 8 hours of incubation in DCA (A) or IA (B) when compared to the control. *Represents p < 0.05.


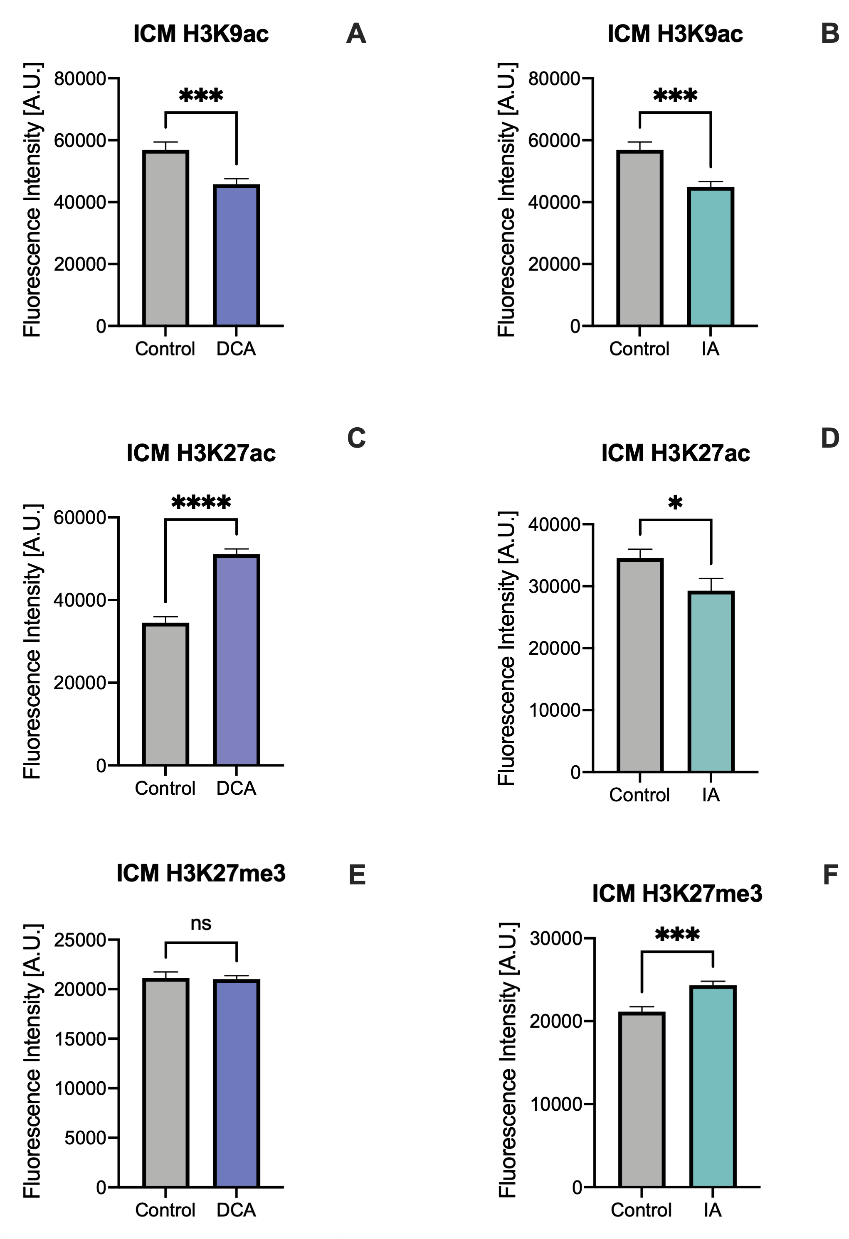


**Supplementary Figure 2** – Fluorescence intensity levels of H3K9ac (A and B), H3K27ac (C and D) and H3K27me3 (E and F) in inner cell masses from the DCA (A, C and E) and IA (B, D and F) groups compared to control group. Nuclei were stained with Hoescht 33342 and immunostained with antibodies against H3K9ac, H3K27ac or H3K27me3. Data are represented as mean ± S.E.M. *Represents p < 0.05. *** Represents p < 0.001. ****Represents p < 0.0001.

**Supplementary table 2 –** Differentially expressed genes (DEGs) of CO vs DCA comparison.

| **GeneID** | **logFC** | **PValue** | **FDR** |
| --- | --- | --- | --- |
| gene:ENSBTAG00000048925 | 3,563310886 | 1,21E-28 | 1,45E-24 |
| CAPN1 | -4,515224764 | 4,35E-15 | 2,60E-11 |
| DNMT3B | -3,560881387 | 2,64E-14 | 1,05E-10 |
| FASN | -2,655899496 | 5,94E-13 | 1,78E-09 |
| gene:ENSBTAG00000045939 | -6,381352611 | 1,19E-12 | 2,86E-09 |
| CTSD | -3,798022001 | 1,57E-12 | 3,14E-09 |
| ZFP36 | -3,248205693 | 6,07E-12 | 1,04E-08 |
| C21H15orf39 | -4,950704441 | 7,55E-12 | 1,13E-08 |
| gene:ENSBTAG00000050274 | 2,287210415 | 1,04E-11 | 1,27E-08 |
| NACC1 | -4,174430948 | 1,06E-11 | 1,27E-08 |
| RBM25 | 2,254925719 | 2,52E-11 | 2,75E-08 |
| COX8B | 9,75928131 | 5,87E-11 | 5,86E-08 |
| HCN2 | -5,435375268 | 6,47E-11 | 5,95E-08 |
| SALL4 | -3,293622837 | 1,02E-10 | 8,72E-08 |
| SOS1 | 2,829496475 | 1,47E-10 | 1,17E-07 |
| ATP5F1D | -3,826890075 | 2,14E-10 | 1,60E-07 |
| ATRX | 2,422560884 | 3,56E-10 | 2,51E-07 |
| PPP4C | -3,858131911 | 4,18E-10 | 2,78E-07 |
| ARHGDIA | -3,192210623 | 4,87E-10 | 3,06E-07 |
| SIN3B | -6,23220031 | 7,20E-10 | 4,30E-07 |
| gene:ENSBTAG00000015551 | -3,326511687 | 1,48E-09 | 8,03E-07 |
| DEK | 2,392434341 | 2,84E-09 | 1,44E-06 |
| MCM2 | -3,321951169 | 2,90E-09 | 1,44E-06 |
| VAT1 | -2,939139962 | 3,47E-09 | 1,66E-06 |
| VDAC2 | 1,464009137 | 3,77E-09 | 1,73E-06 |
| ANP32B | 1,858463259 | 4,13E-09 | 1,83E-06 |
| HSP90AA1 | 1,420628361 | 8,79E-09 | 3,76E-06 |
| 37469 | -3,969766084 | 9,69E-09 | 3,87E-06 |
| gene:ENSBTAG00000019801 | -3,00627594 | 1,75E-08 | 6,76E-06 |
| FBXL19 | -9,342880344 | 2,45E-08 | 9,17E-06 |
| BRB | 5,886865642 | 2,63E-08 | 9,54E-06 |
| HSPE1 | 2,064914324 | 2,73E-08 | 9,59E-06 |
| PRPF38B | 2,321786956 | 2,91E-08 | 9,94E-06 |
| TPT1 | 1,569329717 | 3,65E-08 | 1,21E-05 |
| RCOR2 | -9,273961617 | 4,93E-08 | 1,59E-05 |
| FOXP4 | -2,657756549 | 5,43E-08 | 1,71E-05 |
| TRMT61A | -6,528573792 | 5,78E-08 | 1,77E-05 |
| gene:ENSBTAG00000016748 | -2,804779071 | 7,04E-08 | 2,10E-05 |
| ROCK1 | 2,436192087 | 7,98E-08 | 2,33E-05 |
| PEA15 | -9,220706777 | 8,37E-08 | 2,38E-05 |
| PRRC2A | -2,331011949 | 1,15E-07 | 3,21E-05 |
| SLTM | 2,363914337 | 1,21E-07 | 3,28E-05 |
| FAM222B | -3,797377504 | 1,24E-07 | 3,30E-05 |
| TMSB4X | 1,886253664 | 1,31E-07 | 3,35E-05 |
| TSC22D1_2 | -3,657183089 | 1,33E-07 | 3,35E-05 |
| UNK | -9,172761712 | 1,34E-07 | 3,35E-05 |
| PPIG | 2,463893309 | 1,56E-07 | 3,82E-05 |
| CRIP1 | -2,261417936 | 1,61E-07 | 3,84E-05 |
| CSAD | -9,134686863 | 1,94E-07 | 4,51E-05 |
| UBIAD1 | -4,5219408 | 1,96E-07 | 4,51E-05 |
| ZFAND5 | 1,954819191 | 2,04E-07 | 4,60E-05 |
| LAMA5 | -3,856358933 | 2,39E-07 | 5,30E-05 |
| gene:ENSBTAG00000038258 | -7,060089458 | 2,96E-07 | 6,43E-05 |
| ZNF335 | -5,126636391 | 3,24E-07 | 6,93E-05 |
| EEA1 | 2,102709304 | 3,40E-07 | 7,13E-05 |
| gene:ENSBTAG00000054942 | 2,288180636 | 3,91E-07 | 7,92E-05 |
| ZNF618 | 9,011160242 | 3,90E-07 | 7,92E-05 |
| LAP3 | 2,528466941 | 4,30E-07 | 8,58E-05 |
| PPP2R1A | -2,765854964 | 5,00E-07 | 9,64E-05 |
| RAD50 | 1,874105371 | 4,97E-07 | 9,64E-05 |
| ALG3 | -5,363418671 | 5,24E-07 | 9,75E-05 |
| SMG5 | -2,75649776 | 5,30E-07 | 9,75E-05 |
| TRIM71 | -3,939730866 | 5,25E-07 | 9,75E-05 |
| CD55 | 2,555138045 | 5,49E-07 | 9,94E-05 |
| PRPF40A | 2,028556555 | 5,74E-07 | 0,000102531 |
| PLEC | -2,681012492 | 6,07E-07 | 0,000106732 |
| APOA1 | -2,872686743 | 6,21E-07 | 0,000107626 |
| YIPF2 | -4,885396844 | 6,81E-07 | 0,000116436 |
| ATN1 | -2,019959605 | 7,17E-07 | 0,000119073 |
| TUBB | -1,739511473 | 7,11E-07 | 0,000119073 |
| SRSF11 | 1,79567204 | 7,59E-07 | 0,000124439 |
| SCLT1 | 3,159606662 | 8,83E-07 | 0,000140984 |
| MFAP1 | 2,807591794 | 9,67E-07 | 0,000152227 |
| SHC1 | -2,993218917 | 1,09E-06 | 0,000168642 |
| TMX3 | -4,534476774 | 1,10E-06 | 0,000168642 |
| SMC2 | 1,80635513 | 1,11E-06 | 0,0001688 |
| DDX41 | -5,264940646 | 1,31E-06 | 0,000195391 |
| CYCS | 2,10757041 | 1,38E-06 | 0,00020232 |
| MYL6 | 1,938753752 | 1,39E-06 | 0,00020232 |
| SLC43A1 | -2,705036773 | 1,42E-06 | 0,00020393 |
| CDC34 | -6,890377664 | 1,53E-06 | 0,000215057 |
| NEURL4 | -4,298470597 | 1,85E-06 | 0,000257885 |
| CCDC186 | 1,753359177 | 2,00E-06 | 0,000275563 |
| SLK | 1,962086661 | 2,07E-06 | 0,000281363 |
| BCL2L1 | -3,575623472 | 2,35E-06 | 0,000311824 |
| PNKP | -6,830547384 | 2,49E-06 | 0,000327915 |
| EIF3A | 1,441342665 | 2,79E-06 | 0,000361528 |
| gene:ENSBTAG00000046193 | 5,668016013 | 2,83E-06 | 0,000361528 |
| JUP | -2,25894781 | 2,84E-06 | 0,000361528 |
| PDLIM1 | -1,936466225 | 2,92E-06 | 0,000363439 |
| ZNHIT6 | 2,455912492 | 2,89E-06 | 0,000363439 |
| AP2A1 | -4,74476845 | 3,02E-06 | 0,000371578 |
| TMEM222 | -8,819770363 | 3,04E-06 | 0,000371578 |
| CAMSAP3 | -2,813326135 | 3,12E-06 | 0,000376603 |
| AKT2 | -3,280573058 | 3,19E-06 | 0,000378966 |
| SREK1 | 2,128197456 | 3,20E-06 | 0,000378966 |
| ARHGEF7 | -4,608639637 | 3,55E-06 | 0,000411711 |
| DYRK1B | -8,790516616 | 3,65E-06 | 0,000412261 |
| EIF1AX | 2,391405396 | 3,65E-06 | 0,000412261 |
| LZTFL1 | 3,408115561 | 3,65E-06 | 0,000412261 |
| ROCK2 | 1,795724446 | 3,88E-06 | 0,000429811 |
| SLC27A4 | -3,95734306 | 3,86E-06 | 0,000429811 |
| SEC61G | 2,131440358 | 4,08E-06 | 0,000445775 |
| PIGQ | -4,436348398 | 4,14E-06 | 0,000445831 |
| MYL9 | -2,14066411 | 4,41E-06 | 0,000466601 |
| NISCH | -5,142769664 | 4,38E-06 | 0,000466601 |
| GNL3 | 2,105693753 | 4,55E-06 | 0,000477494 |
| HSPA4 | 1,626354678 | 4,72E-06 | 0,00049093 |
| C29H11orf24 | -4,26032878 | 4,83E-06 | 0,00049783 |
| DNAJA1 | 1,833411505 | 5,14E-06 | 0,000525056 |
| EIF5B | 1,774944078 | 5,47E-06 | 0,000544355 |
| MNT | -4,77309029 | 5,53E-06 | 0,000544355 |
| NES | 2,064483663 | 5,52E-06 | 0,000544355 |
| SH3GL1 | -2,283263964 | 5,54E-06 | 0,000544355 |
| TLE3 | -2,516375699 | 5,55E-06 | 0,000544355 |
| gene:ENSBTAG00000048743 | -8,726165905 | 5,71E-06 | 0,000555075 |
| GATAD2A | -2,052929657 | 6,26E-06 | 0,000598255 |
| TOP2A | 1,646039538 | 6,30E-06 | 0,000598255 |
| CLPTM1L | -3,507993981 | 7,00E-06 | 0,000652337 |
| GOLGB1 | 1,507557508 | 7,03E-06 | 0,000652337 |
| PPP1R13L | -6,703926699 | 7,50E-06 | 0,000683013 |
| SMC5 | 2,563777257 | 7,54E-06 | 0,000683013 |
| CENPF | 1,605253941 | 8,70E-06 | 0,000748447 |
| gene:ENSBTAG00000024999 | -2,979134137 | 8,67E-06 | 0,000748447 |
| gene:ENSBTAG00000050077 | -8,680922488 | 8,66E-06 | 0,000748447 |
| HIST1H2AC | -2,978864206 | 8,51E-06 | 0,000748447 |
| SLC7A4 | -2,129526531 | 8,56E-06 | 0,000748447 |
| TRIM21 | 3,914276382 | 8,47E-06 | 0,000748447 |
| ZBTB40 | -8,684537401 | 8,45E-06 | 0,000748447 |
| DDX46 | 1,587829252 | 8,84E-06 | 0,000755189 |
| MED6 | 3,542374434 | 9,29E-06 | 0,00078796 |
| REXO1 | -3,724916792 | 9,40E-06 | 0,00079172 |
| PPCS | -6,667591058 | 9,48E-06 | 0,000793316 |
| MYADM | -1,651293442 | 9,94E-06 | 0,000825676 |
| SRM | -2,090283212 | 1,02E-05 | 0,000839437 |
| ERF | -2,762467434 | 1,07E-05 | 0,00087344 |
| HSP90B1 | 1,381035289 | 1,29E-05 | 0,001053484 |
| ATG3 | 2,959248335 | 1,33E-05 | 0,001071728 |
| SNX17 | -3,560585488 | 1,34E-05 | 0,001073228 |
| PHC1 | -2,428426574 | 1,36E-05 | 0,001087846 |
| BOP1 | -5,532639922 | 1,39E-05 | 0,001097496 |
| PNN | 1,542239831 | 1,40E-05 | 0,001101728 |
| gene:ENSBTAG00000049055 | 3,302433041 | 1,43E-05 | 0,00110428 |
| PRRC2C | 1,238129451 | 1,45E-05 | 0,00110428 |
| TRIM28 | -1,699121448 | 1,44E-05 | 0,00110428 |
| TRIP11 | 2,097038477 | 1,43E-05 | 0,00110428 |
| SCAP | -2,926936332 | 1,46E-05 | 0,00110462 |
| TP53 | -2,40555673 | 1,50E-05 | 0,001131828 |
| SKI | -3,750053152 | 1,62E-05 | 0,001212351 |
| TRAPPC6A | -8,585921703 | 1,64E-05 | 0,001214626 |
| FTL | -1,359101781 | 1,69E-05 | 0,001234481 |
| GOLGA4 | 1,397075497 | 1,70E-05 | 0,001234481 |
| SMC3 | 1,782984941 | 1,70E-05 | 0,001234481 |
| ERCC2 | -3,354229543 | 1,72E-05 | 0,001235399 |
| MEF2C | 6,969403396 | 1,72E-05 | 0,001235399 |
| LUC7L3 | 1,810730618 | 1,77E-05 | 0,001258968 |
| AKAP9 | 1,60425835 | 1,80E-05 | 0,001271716 |
| ENTPD6 | -8,576957735 | 1,82E-05 | 0,001280297 |
| FMR1 | 1,730818745 | 1,96E-05 | 0,001369429 |
| IK | 1,91829257 | 1,99E-05 | 0,001376098 |
| LGALS3 | 2,283501833 | 1,99E-05 | 0,001376098 |
| EEF2 | -1,442191766 | 2,10E-05 | 0,001442153 |
| gene:ENSBTAG00000023792 | 3,375782155 | 2,18E-05 | 0,001487424 |
| MEGF8 | -8,547605031 | 2,30E-05 | 0,001551809 |
| PTBP1 | -1,430152558 | 2,29E-05 | 0,001551809 |
| SPIC | 1,746545201 | 2,36E-05 | 0,001584804 |
| SAMD4B | -2,601067451 | 2,45E-05 | 0,001636895 |
| LZIC | 3,780632486 | 2,48E-05 | 0,00164645 |
| HNF4A | -1,91064623 | 2,59E-05 | 0,001705635 |
| WDR87 | 4,448737656 | 2,60E-05 | 0,001705635 |
| NEMF | 2,235457712 | 2,61E-05 | 0,001708428 |
| ATG2A | -3,187759157 | 2,65E-05 | 0,001721319 |
| RALY | -2,345083593 | 2,76E-05 | 0,001787177 |
| PSMC2 | 2,007297374 | 2,81E-05 | 0,001807624 |
| ITPRIP | -3,601655303 | 2,86E-05 | 0,001828681 |
| CD3EAP | 2,480980486 | 3,04E-05 | 0,001918293 |
| RIPK2 | 3,071376302 | 3,05E-05 | 0,001918293 |
| gene:ENSBTAG00000008858 | -3,064275685 | 3,24E-05 | 0,002026208 |
| TAGLN2 | -2,029141284 | 3,39E-05 | 0,002114253 |
| BEND3 | -3,300607803 | 3,54E-05 | 0,002192026 |
| CAPN5 | -2,680159923 | 3,59E-05 | 0,002205239 |
| ELMSAN1 | -2,27303691 | 3,59E-05 | 0,002205239 |
| PLXNA1 | -8,464978306 | 3,79E-05 | 0,002287306 |
| UACA | 1,668074953 | 3,77E-05 | 0,002287306 |
| NCL | 1,268484943 | 4,01E-05 | 0,002411808 |
| KMT2D | -1,726688469 | 4,11E-05 | 0,002459919 |
| GTF3C3 | 2,958071359 | 4,58E-05 | 0,002723955 |
| CROT | 4,765772826 | 4,73E-05 | 0,002801029 |
| NAV2 | -2,320169803 | 5,02E-05 | 0,002955314 |
| PEG10 | -5,057968831 | 5,07E-05 | 0,002975224 |
| NFATC2IP | 2,587345166 | 5,13E-05 | 0,002981716 |
| PEX19 | 1,784976609 | 5,13E-05 | 0,002981716 |
| AREL1 | -8,394426557 | 5,36E-05 | 0,003098245 |
| VPS51 | -3,662718327 | 5,43E-05 | 0,003125462 |
| BOLA3 | 4,925207691 | 5,58E-05 | 0,003161227 |
| HCFC1 | -1,733547052 | 5,55E-05 | 0,003161227 |
| RNF40 | -4,260957463 | 5,56E-05 | 0,003161227 |
| TBC1D31 | 2,361592244 | 5,69E-05 | 0,003208215 |
| PXN | -3,570062687 | 5,76E-05 | 0,003237375 |
| DBI | 1,429917 | 5,94E-05 | 0,003309627 |
| WIZ | -3,596193488 | 5,95E-05 | 0,003309627 |
| LRCH4 | -4,088919603 | 6,06E-05 | 0,003358434 |
| FIP1L1 | 1,76130699 | 6,46E-05 | 0,003558739 |
| CLPTM1 | -2,672556155 | 6,50E-05 | 0,003566778 |
| FAM189A1 | -8,369838714 | 6,88E-05 | 0,003759565 |
| HIST1H1E_2 | -3,399933257 | 7,10E-05 | 0,003858179 |
| MBD6 | -3,063537143 | 7,27E-05 | 0,003932468 |
| ECE1 | -2,698387575 | 7,33E-05 | 0,003947669 |
| SH3BP5L | -5,683488625 | 7,67E-05 | 0,004116696 |
| RAN | 1,41307358 | 7,91E-05 | 0,004217932 |
| SCAMP4 | -3,336694208 | 7,93E-05 | 0,004217932 |
| SLC6A8 | -2,268968598 | 8,14E-05 | 0,004290033 |
| EPPK1 | -5,289625026 | 8,26E-05 | 0,004318975 |
| ERRFI1 | -2,175915754 | 8,34E-05 | 0,004338594 |
| COL4A2 | -3,016733137 | 8,45E-05 | 0,004362669 |
| UBR4 | -1,49410096 | 8,46E-05 | 0,004362669 |
| HIC2 | -5,695082722 | 8,51E-05 | 0,004366712 |
| CALM2 | 1,499558288 | 8,61E-05 | 0,004391413 |
| KIRREL1 | -2,532135635 | 8,63E-05 | 0,004391413 |
| ATP13A1 | -3,044204161 | 8,69E-05 | 0,004406985 |
| CSPP1 | 1,982832222 | 8,74E-05 | 0,004413665 |
| SLC7A1 | -8,292004763 | 8,92E-05 | 0,004483866 |
| GGA1 | -3,18031871 | 8,98E-05 | 0,004496632 |
| RBBP7 | 1,890608548 | 9,03E-05 | 0,004501259 |
| COX6B1 | -2,114497962 | 9,22E-05 | 0,004555255 |
| TMC4 | -6,333583127 | 9,25E-05 | 0,004555255 |
| NASP | 1,242494424 | 9,32E-05 | 0,004567779 |
| PRRC2B | -2,144898737 | 9,53E-05 | 0,004653246 |
| RBBP6 | 1,563177173 | 9,70E-05 | 0,004714645 |
| NAPA | -4,560463506 | 9,78E-05 | 0,004718932 |
| SF3B4 | -1,885804126 | 9,78E-05 | 0,004718932 |
| RNF167 | -3,073743426 | 0,000101944 | 0,004897396 |
| ACTN4 | -1,484100662 | 0,000110946 | 0,005308556 |
| ASMTL | -4,534842299 | 0,000112723 | 0,005372078 |
| gene:ENSBTAG00000051741 | 5,974729699 | 0,000116488 | 0,005529481 |
| ASB8 | 2,937098132 | 0,000121994 | 0,005767956 |
| FBLN1 | -3,453422175 | 0,000127677 | 0,005996902 |
| ABCC1 | -3,770894683 | 0,000128502 | 0,006002953 |
| DISP3 | -2,063221549 | 0,000128972 | 0,006002953 |
| COX7B | 1,591419068 | 0,000136849 | 0,006344892 |
| MFSD13A | -3,444621274 | 0,000138435 | 0,006393673 |
| CNTNAP1 | -3,001984871 | 0,000139095 | 0,006399439 |
| ARPC1B | -8,218627359 | 0,00014372 | 0,006536806 |
| SCAPER | 2,202322616 | 0,00014298 | 0,006536806 |
| TMEM94 | -3,224356467 | 0,000143307 | 0,006536806 |
| DGKZ | -3,597414017 | 0,000144984 | 0,006569306 |
| GNPAT | 2,788706676 | 0,000146318 | 0,006604743 |
| ATP5F1C | 1,413596163 | 0,000149981 | 0,00671936 |
| NDUFA5 | 2,490564689 | 0,000149929 | 0,00671936 |
| CETN2 | 2,783118402 | 0,000151705 | 0,006771243 |
| gene:ENSBTAG00000048931 | 2,043549307 | 0,000153657 | 0,006794005 |
| PHKG2 | -8,213227933 | 0,000153919 | 0,006794005 |
| PRKCSH | -3,263377696 | 0,000153145 | 0,006794005 |
| EDF1 | -4,135618369 | 0,000154519 | 0,006795438 |
| FBXW11 | -3,248744466 | 0,000157876 | 0,006892392 |
| PHC3 | 1,947902189 | 0,000157301 | 0,006892392 |
| SLC35E1 | -4,120810546 | 0,00015981 | 0,006951465 |
| PPARD | -3,264866714 | 0,000160794 | 0,006968892 |
| ANO8 | -8,217030757 | 0,000163455 | 0,006983051 |
| PFKFB2 | -8,193921552 | 0,000163428 | 0,006983051 |
| RHOG | -3,747014051 | 0,00016341 | 0,006983051 |
| UPP1 | -4,374420359 | 0,000163021 | 0,006983051 |
| TAX1BP1 | 1,606348136 | 0,000170864 | 0,007222162 |
| NECTIN1 | -2,401962147 | 0,000176223 | 0,00742245 |
| PRMT1 | -2,741094788 | 0,000179966 | 0,007553515 |
| CALD1 | 1,652205327 | 0,000186297 | 0,00779192 |
| RADX | 2,416993563 | 0,000188956 | 0,007875592 |
| CCNF | -4,45325695 | 0,00019184 | 0,007940461 |
| UBE2S | -3,492562187 | 0,000191646 | 0,007940461 |
| ZFC3H1 | 1,528416475 | 0,000203514 | 0,008394604 |
| LRP1 | -5,534234948 | 0,000204909 | 0,008423091 |
| BACE1 | -8,153867009 | 0,000208685 | 0,00851978 |
| MYO1C | -1,864037534 | 0,000208279 | 0,00851978 |
| CSTB_1 | 1,4104444 | 0,000210348 | 0,008555368 |
| SARNP | 2,205233472 | 0,000210988 | 0,008555368 |
| SLC34A2 | -2,925532938 | 0,000212299 | 0,008579456 |
| DNTTIP2 | 1,553603526 | 0,000216725 | 0,008709687 |
| KCNC3 | -4,86929718 | 0,000216978 | 0,008709687 |
| TSKU | -3,710627722 | 0,00021787 | 0,008716274 |
| TAF10 | 2,129990341 | 0,000219187 | 0,008739722 |
| GMPR2 | -8,110706209 | 0,000220507 | 0,00876313 |
| ITGA5 | -1,385632079 | 0,000222422 | 0,008809961 |
| EIF3E | 1,537528018 | 0,000225174 | 0,008831253 |
| LYSMD2 | 4,468024944 | 0,000223898 | 0,008831253 |
| NR1H2 | -2,508434511 | 0,000224699 | 0,008831253 |
| gene:ENSBTAG00000052021 | 6,391329399 | 0,000236137 | 0,009230949 |
| DIS3L | 2,709232246 | 0,000239079 | 0,009315512 |
| NAA11 | -8,116666312 | 0,00024238 | 0,009382997 |
| PRSS16 | -8,117432444 | 0,00024178 | 0,009382997 |
| gene:ENSBTAG00000026119 | -1,727084329 | 0,00024702 | 0,009519145 |
| PCBP1 | -1,850743445 | 0,000247488 | 0,009519145 |
| EIF3M | 1,730141434 | 0,000253349 | 0,009651474 |
| GLUL | -1,45011221 | 0,000252702 | 0,009651474 |
| PFAS | -3,048115143 | 0,000252711 | 0,009651474 |
| CACYBP | 1,791184309 | 0,000255057 | 0,009685677 |
| ATP5MC2 | -2,082483758 | 0,000264196 | 0,009969422 |
| TNRC6C | -1,964280644 | 0,000263744 | 0,009969422 |
| PSIP1 | 1,603025526 | 0,000268925 | 0,010115975 |
| WDR77 | 1,989948487 | 0,000272097 | 0,010171332 |
| CCAR1 | 1,472314964 | 0,000274151 | 0,010184471 |
| RAD54L2 | -2,095237969 | 0,000273343 | 0,010184471 |
| DPF2 | -1,864254593 | 0,000277774 | 0,01028711 |
| MORC3 | 2,172633379 | 0,000279343 | 0,010313269 |
| gene:ENSBTAG00000049312 | 2,67844061 | 0,00028321 | 0,010423887 |
| KIF20B | 2,138197452 | 0,000284485 | 0,010438686 |
| gene:ENSBTAG00000022275 | 2,87238282 | 0,000287001 | 0,010498807 |
| gene:ENSBTAG00000017939 | -2,120859416 | 0,000298297 | 0,010878741 |
| SASS6 | 1,979709429 | 0,000300239 | 0,010916271 |
| C2CD2 | -5,082435569 | 0,000304633 | 0,011042478 |
| LYPLA2 | -2,999051481 | 0,000310489 | 0,011220741 |
| GRAMD1A | -2,595798207 | 0,00031686 | 0,011382811 |
| SMC1A | 1,481990317 | 0,000316876 | 0,011382811 |
| gene:ENSBTAG00000019309 | 3,592093444 | 0,000318215 | 0,011396685 |
| SHARPIN | -3,237086784 | 0,000341667 | 0,01220006 |
| DHCR7 | -2,468244369 | 0,000345148 | 0,012287664 |
| TOP1 | 1,91563451 | 0,000352642 | 0,012517232 |
| TBC1D10A | -8,021343377 | 0,000354877 | 0,012559302 |
| IFT20 | 2,519320218 | 0,000365945 | 0,012912784 |
| CDCA5 | -5,440471909 | 0,000369019 | 0,012944875 |
| LIG4 | 3,648735338 | 0,000372176 | 0,013017453 |
| gene:ENSBTAG00000050561 | 3,247588232 | 0,000373594 | 0,013028964 |
| ATP5MC1 | -2,164472849 | 0,000380344 | 0,013168644 |
| CSRNP1 | -3,076841406 | 0,000382003 | 0,013168644 |
| gene:ENSBTAG00000031205 | -4,838043529 | 0,000380978 | 0,013168644 |
| TRMT2A | -3,008872381 | 0,000380294 | 0,013168644 |
| AEN | -8,011150107 | 0,000385431 | 0,013248624 |
| MARK2 | -2,871504355 | 0,000394428 | 0,013481173 |
| PPT1 | 1,689872777 | 0,00039445 | 0,013481173 |
| PGLS | -5,007002213 | 0,000418718 | 0,014269814 |
| ADH5 | 1,911986138 | 0,00042842 | 0,014517722 |
| ATP1B3 | 1,382593219 | 0,000439839 | 0,014834907 |
| gene:ENSBTAG00000002290 | -3,149694981 | 0,00044026 | 0,014834907 |
| KMT2E | 1,452177973 | 0,000448676 | 0,015033788 |
| SUZ12 | 1,557989203 | 0,000448078 | 0,015033788 |
| FZR1 | -4,363693078 | 0,000451283 | 0,015078889 |
| ERBB2 | -2,663509639 | 0,000452747 | 0,015085683 |
| RPTOR | -2,379397264 | 0,000454784 | 0,015111472 |
| FIS1 | -3,086294855 | 0,000460056 | 0,01524428 |
| NKAPD1 | 2,483998982 | 0,000462532 | 0,015283985 |
| gene:ENSBTAG00000049344 | 2,780465071 | 0,000464639 | 0,015311328 |
| DENND4B | -7,955522862 | 0,000470684 | 0,015425524 |
| PELP1 | -4,333748062 | 0,000470568 | 0,015425524 |
| AIF1L | -2,696444072 | 0,00047456 | 0,015510067 |
| BCOR | -1,413980447 | 0,000493049 | 0,016026775 |
| PI4KA | -2,328908686 | 0,000491734 | 0,016026775 |
| DCAF15 | -3,774872763 | 0,000497813 | 0,016137789 |
| GSE1 | -2,627934466 | 0,000501213 | 0,016204086 |
| FAM13B | 1,961017688 | 0,000502631 | 0,016206123 |
| EIF4H | -1,361337158 | 0,00050607 | 0,016273131 |
| ATP5PF | 1,753506808 | 0,000510082 | 0,016358164 |
| NDUFA13 | -2,520513203 | 0,000513514 | 0,016424211 |
| CENPE | 1,575546017 | 0,000517288 | 0,016500812 |
| SAT1 | 2,71767027 | 0,000520624 | 0,01651912 |
| SLC7A5 | -1,842756373 | 0,00052031 | 0,01651912 |
| PTGFRN | -3,360169491 | 0,000524675 | 0,016603592 |
| ZCCHC17 | 1,783464559 | 0,000529631 | 0,016716207 |
| YTHDC1 | 1,66829106 | 0,000533138 | 0,016782627 |
| APOE | -7,916400174 | 0,000546794 | 0,01707768 |
| PLD3 | -1,659940957 | 0,000546349 | 0,01707768 |
| TMEM109 | -2,15183784 | 0,000546627 | 0,01707768 |
| MLH3 | 4,539487635 | 0,000551675 | 0,017140611 |
| RNF44 | -1,918129612 | 0,000550448 | 0,017140611 |
| CALCOCO1 | 1,702924228 | 0,000567491 | 0,017540911 |
| FAM84B | 1,553824697 | 0,000567145 | 0,017540911 |
| gene:ENSBTAG00000050846 | -7,902189837 | 0,000579999 | 0,017802239 |
| GNG4 | -4,269152816 | 0,000579414 | 0,017802239 |
| MYBBP1A | -1,521044297 | 0,000580411 | 0,017802239 |
| HTT | -2,299261206 | 0,000584917 | 0,017894565 |
| gene:ENSBTAG00000007187 | -4,139183144 | 0,000593374 | 0,018097649 |
| ITGB4 | -7,898400706 | 0,000594581 | 0,018097649 |
| SHANK2 | -2,869850553 | 0,000596553 | 0,018111577 |
| CFAP36 | 2,643917876 | 0,000600924 | 0,018198094 |
| DCAF1 | 1,54751864 | 0,000612849 | 0,018465746 |
| GLI3 | -3,080561948 | 0,000612389 | 0,018465746 |
| CDC16 | 1,928064529 | 0,000623532 | 0,018711757 |
| gene:ENSBTAG00000019545 | -5,343046877 | 0,000624142 | 0,018711757 |
| IGSF9B | -4,666474048 | 0,000628941 | 0,01880849 |
| ZNF638 | 1,395380475 | 0,000631856 | 0,018848534 |
| gene:ENSBTAG00000027610 | 1,937971386 | 0,000635627 | 0,018866938 |
| THOC2 | 1,653220194 | 0,000635133 | 0,018866938 |
| ZC3H3 | -7,887103943 | 0,000641506 | 0,018994296 |
| CDH2 | -2,902547784 | 0,000648056 | 0,019093715 |
| ZC3H15 | 1,506865437 | 0,000646859 | 0,019093715 |
| DLG5 | -1,85779163 | 0,000650173 | 0,019109009 |
| MED4 | 2,447796353 | 0,000651809 | 0,019110134 |
| EMC1 | -2,837334227 | 0,000658241 | 0,019111362 |
| ESPL1 | -1,933561048 | 0,000658221 | 0,019111362 |
| NPBWR1 | -5,970027839 | 0,000658195 | 0,019111362 |
| RNF13 | 2,432338432 | 0,000660817 | 0,019139701 |
| DNAJC3 | 1,862140578 | 0,000672955 | 0,019444177 |
| JPT2 | -2,603679464 | 0,000684487 | 0,019729717 |
| C20H5orf51 | 1,699469782 | 0,000690271 | 0,019848621 |
| MINAR1 | -5,968888284 | 0,000696972 | 0,019945393 |
| SHKBP1 | -4,913022339 | 0,000695999 | 0,019945393 |
| FHL3 | -7,838317058 | 0,000728792 | 0,020756689 |
| P4HB | -1,660276462 | 0,000736264 | 0,020915074 |
| SLC25A37 | -4,005449732 | 0,00073785 | 0,020915074 |
| NDUFV1 | -1,773833143 | 0,000744204 | 0,021045324 |
| ATR | 2,085340503 | 0,000750964 | 0,021186391 |
| DUSP4 | -7,843505154 | 0,000755588 | 0,021266682 |
| NR4A3 | -7,824203208 | 0,000771366 | 0,021609098 |
| MFGE8 | -2,416897088 | 0,000775385 | 0,021670931 |
| CORO1B | -7,822163485 | 0,00078118 | 0,021712005 |
| SETD1A | -2,066836438 | 0,0007823 | 0,021712005 |
| ZNF143 | 2,637882532 | 0,000779875 | 0,021712005 |
| SHF | -3,269169636 | 0,000792411 | 0,021941719 |
| KDM6A | 1,294783237 | 0,000799256 | 0,021978617 |
| PDP2 | -2,271533607 | 0,000798921 | 0,021978617 |
| SLC4A11 | -4,080507576 | 0,000798946 | 0,021978617 |
| MNS1 | 5,117680951 | 0,000801156 | 0,021980349 |
| CUL9 | -2,84085216 | 0,000804155 | 0,022012124 |
| TIAM2 | -7,836198922 | 0,000806136 | 0,022015976 |
| gene:ENSBTAG00000051208 | 2,463397219 | 0,000808155 | 0,022020851 |
| RBX1 | 1,82416761 | 0,000815851 | 0,022180035 |
| TTLL4 | -2,200101972 | 0,000824644 | 0,022368234 |
| TNKS1BP1 | -4,620561682 | 0,000832348 | 0,022475286 |
| ZCCHC14 | -7,821313492 | 0,000832147 | 0,022475286 |
| ADAMTS7 | -7,804074423 | 0,00086738 | 0,023344837 |
| ARHGAP33 | -7,808274054 | 0,000869264 | 0,023344837 |
| HGH1 | -2,300604087 | 0,000872358 | 0,023344837 |
| HSPG2 | -3,469547116 | 0,000872122 | 0,023344837 |
| SVBP | 3,198540078 | 0,000874404 | 0,023347369 |
| ATG7 | -4,586248332 | 0,000886019 | 0,023458062 |
| ESD | 2,115009502 | 0,000885987 | 0,023458062 |
| MGC148714 | 1,417983916 | 0,000886394 | 0,023458062 |
| SERINC3 | -7,814732166 | 0,000883359 | 0,023458062 |
| AKT1 | -5,293558206 | 0,000899512 | 0,02375269 |
| WDR5 | -2,918919643 | 0,000906601 | 0,023887149 |
| CPNE3 | -1,468258834 | 0,000930151 | 0,024400147 |
| PNRC1 | -1,682140903 | 0,000930027 | 0,024400147 |
| CHSY3 | 6,229549547 | 0,000932387 | 0,024405277 |
| PRR12 | -3,201213695 | 0,000950395 | 0,024822332 |
| PLXNB2 | -1,469498167 | 0,000957922 | 0,024964401 |
| TCF19 | -7,771842017 | 0,000962845 | 0,025038152 |
| gene:ENSBTAG00000048800 | -5,905666946 | 0,000968128 | 0,025120916 |
| SAP30L | 2,188764055 | 0,000980825 | 0,025395291 |
| MMP15 | -3,270341043 | 0,000984048 | 0,025423718 |
| CCHCR1 | 2,330844811 | 0,000991019 | 0,025493699 |
| RARA | -4,39627601 | 0,000990621 | 0,025493699 |
| CALR | 1,289875749 | 0,001010454 | 0,025882337 |
| HNRNPH3 | 1,302945531 | 0,001008687 | 0,025882337 |
| gene:ENSBTAG00000024578 | 1,952847912 | 0,001028847 | 0,026297148 |
| CAD | -1,967016683 | 0,001031887 | 0,026318627 |
| PEG3 | 1,523212851 | 0,001037614 | 0,026408378 |
| ARMH3 | 2,09414782 | 0,001042481 | 0,026437072 |
| FOXRED2 | -3,539350155 | 0,001045372 | 0,026437072 |
| SLC35C2 | -7,754140214 | 0,001044277 | 0,026437072 |
| GGT5 | -7,739494608 | 0,001054893 | 0,026565543 |
| LTBR | -7,750592374 | 0,001053934 | 0,026565543 |
| KPNA6 | -5,905616622 | 0,001059069 | 0,026614683 |
| JAK1 | 1,560148708 | 0,001074986 | 0,026958032 |
| HIST1H2BN | -1,664341673 | 0,001079722 | 0,027020156 |
| HNRNPA1 | 0,996483484 | 0,001096266 | 0,027376895 |
| SOWAHB | -5,899699529 | 0,001105112 | 0,027540313 |
| PHOSPHO1 | -3,212982301 | 0,001109207 | 0,02758489 |
| CLSTN1 | -3,068225183 | 0,001128195 | 0,027998897 |
| DNAJB6 | -2,714132115 | 0,001130997 | 0,028010318 |
| SUPT4H1 | 2,899914885 | 0,001146707 | 0,02834073 |
| CHCHD2 | 1,183883797 | 0,001152083 | 0,028414892 |
| RNASEH2A | -2,076926089 | 0,001173759 | 0,028830597 |
| DOHH | -7,726790651 | 0,001185238 | 0,028950854 |
| MET | 2,3173862 | 0,001193191 | 0,028950854 |
| NSRP1 | 1,698386772 | 0,001195596 | 0,028950854 |
| PLGRKT | 2,888939557 | 0,001187344 | 0,028950854 |
| SCRIB | -7,728751624 | 0,001188958 | 0,028950854 |
| SNX33 | -3,197411965 | 0,001191718 | 0,028950854 |
| CWC27 | 2,233219981 | 0,00119907 | 0,028976325 |
| WAPL | 1,296168089 | 0,001204629 | 0,029051961 |
| CCT8 | 1,258263098 | 0,001209462 | 0,029059069 |
| FURIN | -3,906702523 | 0,001211184 | 0,029059069 |
| NSMCE4A | 2,206307201 | 0,001212212 | 0,029059069 |
| SPDL1 | 2,368904451 | 0,001224451 | 0,029293776 |
| CHTF18 | -2,617192115 | 0,001236753 | 0,029421208 |
| HMOX1 | -2,634098384 | 0,001233409 | 0,029421208 |
| LEO1 | 1,697397143 | 0,001237157 | 0,029421208 |
| gene:ENSBTAG00000053316 | 2,745992154 | 0,001248498 | 0,029632017 |
| ANXA11 | 1,510331164 | 0,001257186 | 0,029779115 |
| BOK | -7,695853816 | 0,001261324 | 0,029818108 |
| GLYR1 | -1,959367678 | 0,001274025 | 0,030046482 |
| PKP3 | -7,698488345 | 0,001276008 | 0,030046482 |
| CA4 | -5,206938416 | 0,001286126 | 0,030165961 |
| RANBP1 | -2,131803816 | 0,001285865 | 0,030165961 |
| MOSPD2 | 3,075135358 | 0,001304703 | 0,030541794 |
| MTDH | 1,295416859 | 0,001310671 | 0,030561889 |
| WAC | 1,170513079 | 0,001309085 | 0,030561889 |
| gene:ENSBTAG00000055145 | -2,817636345 | 0,001316604 | 0,030640508 |
| SETDB2 | 2,650727676 | 0,001330528 | 0,030904424 |
| L1CAM | -4,556575313 | 0,001334487 | 0,030936306 |
| GGTA1 | 1,454355202 | 0,001343896 | 0,030974333 |
| GNA13 | -2,576841723 | 0,001340068 | 0,030974333 |
| TBL3 | -4,002870356 | 0,001343696 | 0,030974333 |
| CD2AP | 1,189120577 | 0,001366253 | 0,031268805 |
| CELF1 | -2,52607675 | 0,001370557 | 0,031268805 |
| gene:ENSBTAG00000049577 | -7,699888915 | 0,001372356 | 0,031268805 |
| RIN3 | -1,676934014 | 0,001369558 | 0,031268805 |
| SLC12A9 | -5,815854633 | 0,001361768 | 0,031268805 |
| THADA | -3,122820562 | 0,001362191 | 0,031268805 |
| CD63 | -2,82029246 | 0,001382315 | 0,031376193 |
| SPRY2 | -2,337443893 | 0,00138114 | 0,031376193 |
| NFRKB | -4,026441969 | 0,001389735 | 0,031425348 |
| POLR2L | -2,339769978 | 0,001388863 | 0,031425348 |
| STXBP1 | 1,691730368 | 0,001413203 | 0,031895716 |
| PTPRS | -4,805406628 | 0,001419638 | 0,031929936 |
| LLGL2 | -3,23959956 | 0,001429221 | 0,032075691 |
| RAVER1 | -2,076182043 | 0,001435273 | 0,032151199 |
| MED29 | -4,153855272 | 0,001448351 | 0,032383503 |
| GPAA1 | -2,678851496 | 0,001466318 | 0,032715834 |
| KRT18 | 1,334047048 | 0,001468684 | 0,032715834 |
| MARVELD1 | -3,283457967 | 0,001474398 | 0,032782068 |
| BCL9L | -3,844215986 | 0,001479815 | 0,032841456 |
| PHF20 | 1,732701877 | 0,001483705 | 0,032866804 |
| RTN2 | -7,659768532 | 0,001504194 | 0,033259091 |
| PCDHGA8 | -3,017430005 | 0,001513363 | 0,033400093 |
| UBTD1 | -7,630219613 | 0,001517286 | 0,03342501 |
| FNBP1L | 1,353192493 | 0,001520919 | 0,033443433 |
| gene:ENSBTAG00000014417 | -2,934084981 | 0,001531557 | 0,033553995 |
| gene:ENSBTAG00000050847 | 2,191173554 | 0,001530327 | 0,033553995 |
| PHF13 | -7,630403721 | 0,001550102 | 0,033898204 |
| FNBP4 | 1,29273973 | 0,001556124 | 0,033905937 |
| MICAL2 | -4,507900304 | 0,001554002 | 0,033905937 |
| SCAF11 | 1,306059472 | 0,001583077 | 0,034430489 |
| COMMD1 | 1,941336847 | 0,001593754 | 0,034599784 |
| ACTR3 | 1,26534246 | 0,001600096 | 0,034674538 |
| gene:ENSBTAG00000019479 | -7,629640162 | 0,001633521 | 0,035334865 |
| TXNDC9 | 3,234755813 | 0,001649852 | 0,035623704 |
| SYNRG | -2,531679636 | 0,001658972 | 0,035756073 |
| ALDH16A1 | -4,124376285 | 0,001668862 | 0,035904544 |
| WNK2 | -3,936261408 | 0,001677958 | 0,036035424 |
| FHL2 | 3,187678297 | 0,001681016 | 0,036036396 |
| DAAM1 | 1,664767313 | 0,001700466 | 0,036323163 |
| VBP1 | 1,824680754 | 0,001699009 | 0,036323163 |
| SMG9 | -2,944742009 | 0,001706153 | 0,036379673 |
| SCNN1A | -2,225651084 | 0,001718955 | 0,036522454 |
| ZNF385A | -7,588654913 | 0,001717696 | 0,036522454 |
| TLK2 | 1,695619309 | 0,001725515 | 0,036596825 |
| ARSB | -3,430885002 | 0,001737247 | 0,036715458 |
| DGCR8 | -2,553002437 | 0,001735327 | 0,036715458 |
| ENTPD5 | 5,094293395 | 0,001747886 | 0,036875159 |
| CNNM2 | -3,084609238 | 0,001761907 | 0,037105505 |
| NEDD4L | 2,42943918 | 0,00176877 | 0,037184578 |
| STARD3 | -5,122455914 | 0,00177941 | 0,037277663 |
| ARID4B | 1,713173995 | 0,001792481 | 0,03745667 |
| COX7A2 | 1,294904199 | 0,001797369 | 0,03745667 |
| KLF6 | -1,228935049 | 0,001796302 | 0,03745667 |
| ZC3H10 | 1,797012166 | 0,001808469 | 0,037622439 |
| RNF26 | -4,277278024 | 0,001814304 | 0,037678301 |
| ABCF3 | -4,10901519 | 0,001828611 | 0,037909603 |
| PSRC1 | -2,695349201 | 0,001837379 | 0,038025487 |
| HHEX | 4,861428555 | 0,001845787 | 0,038111749 |
| PCYT2 | -2,494172494 | 0,00184792 | 0,038111749 |
| BSG | -1,105210491 | 0,001857065 | 0,03821697 |
| CNPPD1 | -2,597788383 | 0,001862606 | 0,03821697 |
| MYO10 | -3,818422855 | 0,001860598 | 0,03821697 |
| ST14 | -2,654089525 | 0,001890837 | 0,03872979 |
| DUSP14 | 1,900402211 | 0,001900324 | 0,03873466 |
| EXOSC6 | -4,454727022 | 0,001900542 | 0,03873466 |
| NUDT16L1 | -5,751398116 | 0,001904028 | 0,03873466 |
| PPRC1 | -1,258985626 | 0,001902414 | 0,03873466 |
| RNF4 | -1,530214241 | 0,001924371 | 0,039082054 |
| TAF6 | -4,097661541 | 0,001932757 | 0,039185835 |
| FKBP8 | -3,303084812 | 0,001943663 | 0,039340265 |
| USP53 | 1,464194522 | 0,001976121 | 0,039929667 |
| CDK18 | -5,084483341 | 0,002025725 | 0,040288494 |
| CPSF6 | -1,654693694 | 0,001997835 | 0,040288494 |
| CRTC2 | -2,385766664 | 0,002014745 | 0,040288494 |
| ENO1 | -1,293840924 | 0,002012874 | 0,040288494 |
| gene:ENSBTAG00000027426 | 1,386873317 | 0,002005944 | 0,040288494 |
| MICU2 | 1,671880289 | 0,002017686 | 0,040288494 |
| MMS19 | -2,334083888 | 0,00201161 | 0,040288494 |
| MRTFA | -2,056567703 | 0,002021518 | 0,040288494 |
| PER2 | -7,547282696 | 0,002004868 | 0,040288494 |
| SYF2 | 1,621144569 | 0,00202756 | 0,040288494 |
| FOLR3 | -7,543357899 | 0,002040445 | 0,040410272 |
| NIPBL | 1,151035584 | 0,002037976 | 0,040410272 |
| ATAD1 | 1,625737473 | 0,002056384 | 0,040563777 |
| NOP58 | 1,086624465 | 0,002058369 | 0,040563777 |
| PPP2R5B | -7,535836358 | 0,002051642 | 0,040563777 |
| B4GALNT3 | -4,214824287 | 0,002064836 | 0,040602684 |
| TCF3 | -2,016237076 | 0,002067132 | 0,040602684 |
| USP48 | 0,98888471 | 0,002088357 | 0,040952346 |
| EIF2S2 | 1,274999284 | 0,00209767 | 0,04100804 |
| gene:ENSBTAG00000011437 | -2,067633203 | 0,002098054 | 0,04100804 |
| TOM1 | -5,711011928 | 0,002108335 | 0,041141768 |
| PARP11 | 3,272995172 | 0,002138232 | 0,041657218 |
| MPHOSPH8 | 1,413116742 | 0,002151928 | 0,04185587 |
| gene:ENSBTAG00000047550 | 1,409542099 | 0,002159345 | 0,04193196 |
| CDCA2 | 1,715355194 | 0,002174002 | 0,042148149 |
| gene:ENSBTAG00000053518 | 4,236499744 | 0,002188517 | 0,042279273 |
| RBBP8 | 1,596213361 | 0,002191368 | 0,042279273 |
| TAX1BP3 | -2,31852849 | 0,002188222 | 0,042279273 |
| LONP1 | -1,856165861 | 0,00220761 | 0,042524035 |
| RAF1 | -2,263980572 | 0,002215503 | 0,042607475 |
| gene:ENSBTAG00000052305 | 2,381866169 | 0,002231673 | 0,042849554 |
| WBP4 | 2,25743228 | 0,002255307 | 0,043233954 |
| KANSL3 | -3,191895773 | 0,002260101 | 0,043256519 |
| DAB2 | 1,522256639 | 0,002284535 | 0,043640621 |
| FBH1 | -2,314473228 | 0,002287466 | 0,043640621 |
| TFIP11 | -2,969007723 | 0,002308406 | 0,04396999 |
| FAM189B | -7,508032188 | 0,002325519 | 0,044225522 |
| UBE2V2 | 1,897232666 | 0,002331875 | 0,044276023 |
| PCMTD2 | -2,867108956 | 0,002337793 | 0,044318042 |
| GRTP1 | -7,492235586 | 0,002355677 | 0,044586398 |
| BLOC1S2 | 2,127026087 | 0,002379243 | 0,044821912 |
| R3HDM2 | 1,30667444 | 0,00237571 | 0,044821912 |
| SIMC1 | -2,32735245 | 0,002379361 | 0,044821912 |
| FMR1NB | 4,66021603 | 0,002413519 | 0,045393891 |
| ALDOA | -1,222844954 | 0,00241901 | 0,045425744 |
| FAM83H | -2,043681596 | 0,002428629 | 0,045534899 |
| AHI1 | 1,883331064 | 0,002440879 | 0,045692958 |
| NKRF | 3,003456542 | 0,002462658 | 0,046028619 |
| ZBTB49 | -2,959238257 | 0,002477049 | 0,046225365 |
| PHF14 | 3,174838152 | 0,002489389 | 0,046311148 |
| ZMYND19 | -4,048185244 | 0,002486159 | 0,046311148 |
| DENND6A | 2,415186603 | 0,002504716 | 0,046523931 |
| FBXO6 | -7,483835255 | 0,002516075 | 0,046594295 |
| WDR55 | 1,706686409 | 0,002516294 | 0,046594295 |
| gene:ENSBTAG00000053621 | -2,416690626 | 0,002543954 | 0,047033665 |
| E2F1 | -4,414721717 | 0,002586936 | 0,047632896 |
| TMEM37 | -3,492698755 | 0,002582083 | 0,047632896 |
| UNC5B | -2,915446244 | 0,002588312 | 0,047632896 |
| GAREM1 | -3,903757107 | 0,002610412 | 0,047892244 |
| SERAC1 | 2,371709032 | 0,002620273 | 0,047999552 |
| SH3RF1 | -5,678417674 | 0,002657223 | 0,048601996 |
| JUNB | -2,496829574 | 0,002665337 | 0,048675974 |
| LARS2 | -4,366414187 | 0,00267893 | 0,048849637 |
| NNAT | -4,682763908 | 0,002685562 | 0,048896029 |
| CHERP | -1,540313948 | 0,002692105 | 0,04894066 |
| DOT1L | -2,041426536 | 0,002699226 | 0,048961011 |
| HTATSF1 | 4,293407488 | 0,00270141 | 0,048961011 |
| CELSR1 | -4,663360827 | 0,002707552 | 0,048998088 |
| TBRG4 | -1,855846331 | 0,002713532 | 0,049032125 |
| BOD1L1 | 1,050300501 | 0,002729017 | 0,049082907 |
| PLBD2 | -3,453115969 | 0,002732755 | 0,049082907 |
| SLC27A3 | -7,457517437 | 0,002729238 | 0,049082907 |
| ZC3H4 | -5,033006477 | 0,002726256 | 0,049082907 |
| ITSN1 | 1,273594788 | 0,002737706 | 0,049098106 |
| LIMK2 | -2,568658508 | 0,00275312 | 0,049300626 |
| CEP152 | 1,714244808 | 0,002761587 | 0,049304635 |
| SEPT11 | 1,028868954 | 0,002758524 | 0,049304635 |
| YARS | -2,266233861 | 0,002773859 | 0,049449923 |
| HMGA1 | -1,178170091 | 0,002790563 | 0,049673678 |
| SFN | -1,587435395 | 0,002799273 | 0,049731585 |
| TUBB4A | -7,412561521 | 0,002802131 | 0,049731585 |
| UBN2 | 1,309082244 | 0,00282625 | 0,050085329 |
| TAOK3 | 1,430257598 | 0,002832711 | 0,050125567 |
| CC2D1B | -3,378084743 | 0,002863787 | 0,050600613 |
| ADSS | 1,853113079 | 0,002892549 | 0,050693124 |
| FAM160A2 | -2,590859085 | 0,002892867 | 0,050693124 |
| gene:ENSBTAG00000003367 | -2,492306797 | 0,002894449 | 0,050693124 |
| HNRNPD | 1,044426276 | 0,002876832 | 0,050693124 |
| RAD23A | -2,774836626 | 0,002889625 | 0,050693124 |
| SALL1 | -3,254629546 | 0,002883572 | 0,050693124 |
| ZNF296 | -2,011277035 | 0,002906774 | 0,050834547 |
| CCND3 | -2,546904748 | 0,002920727 | 0,05100399 |
| GPC1 | -3,563685373 | 0,00293624 | 0,051200153 |
| BAZ1A | 1,419642261 | 0,002959085 | 0,051411457 |
| SCEL | 2,732810666 | 0,002956201 | 0,051411457 |
| SLC25A11 | -3,609327204 | 0,002961252 | 0,051411457 |
| PRELID3B | 1,574908699 | 0,002982562 | 0,051706395 |
| RBM6 | 1,556614317 | 0,003017166 | 0,052230596 |
| NME2 | 1,061569329 | 0,003025176 | 0,052293579 |
| MMP14 | -2,155501826 | 0,003045755 | 0,052573331 |
| SMIM3 | 3,021611271 | 0,003061865 | 0,052699331 |
| TPM1 | 1,293786546 | 0,003060043 | 0,052699331 |
| C6H4orf3 | 2,155884763 | 0,003073851 | 0,052829614 |
| MARK4 | -3,83477847 | 0,003094741 | 0,0528847 |
| MAX | -3,209671646 | 0,003088221 | 0,0528847 |
| PHLDB2 | 2,017830339 | 0,00308204 | 0,0528847 |
| MED9 | -7,387265168 | 0,003100442 | 0,052906552 |
| ADAP1 | -7,43249151 | 0,003111696 | 0,05294752 |
| DIS3 | 1,812145236 | 0,003109728 | 0,05294752 |
| SLC2A8 | 3,025795556 | 0,003136795 | 0,053298783 |
| PCNX3 | -1,668468206 | 0,003150591 | 0,053457257 |
| DTX2 | -3,73723954 | 0,003163997 | 0,053608689 |
| RPF1 | 2,237442215 | 0,003184643 | 0,053882175 |
| ACVR2B | -3,298100015 | 0,003189657 | 0,053890779 |
| ATXN2L | -1,282580418 | 0,003200846 | 0,053927489 |
| PCNA | 1,436467979 | 0,003200672 | 0,053927489 |
| VPS18 | -5,592059293 | 0,003211976 | 0,054038897 |
| SLC25A1 | -2,879536575 | 0,003221608 | 0,05412482 |
| ENDOG | -7,382793963 | 0,003258385 | 0,054576794 |
| NR1D2 | -2,828905521 | 0,003269196 | 0,054576794 |
| PAWR | 2,766826809 | 0,003271323 | 0,054576794 |
| RAD54B | 3,195300196 | 0,003271078 | 0,054576794 |
| TRIM11 | -7,389920136 | 0,003266491 | 0,054576794 |
| PFDN5 | 1,521928662 | 0,003285419 | 0,054735635 |
| ABAT | -7,365838038 | 0,003294628 | 0,054736588 |
| MTOR | -1,607143884 | 0,003292341 | 0,054736588 |
| SRRD | -7,358073517 | 0,003300938 | 0,054765349 |
| FBXO22 | 2,790054561 | 0,003311872 | 0,054856739 |
| RTRAF | 1,376082617 | 0,003315618 | 0,054856739 |
| MTERF4 | 2,106474486 | 0,00334272 | 0,055228755 |
| NDUFS5 | 1,65784995 | 0,003351144 | 0,055291568 |
| HSPD1_1 | 0,95888228 | 0,003356429 | 0,055302477 |
| MEF2D | -2,331175224 | 0,003379838 | 0,055611583 |
| DEPDC7 | 2,525216875 | 0,003394245 | 0,055771921 |
| FLNA | -1,322792109 | 0,003432728 | 0,056249711 |
| PCDH1 | -1,614444408 | 0,003429451 | 0,056249711 |
| RNF20 | 1,740883077 | 0,003469276 | 0,056693274 |
| TMEM110 | -3,071998614 | 0,003465773 | 0,056693274 |
| PUS7L | 3,2311212 | 0,003475091 | 0,056710836 |
| PCIF1 | -4,356230598 | 0,003482293 | 0,056750943 |
| ELF2 | 1,276746074 | 0,003500992 | 0,056978053 |
| SLITRK5 | -2,997468595 | 0,003506865 | 0,056996083 |
| ERCC4 | -2,749165206 | 0,003515514 | 0,057059137 |
| CFAP410 | -7,353504125 | 0,003528487 | 0,057065785 |
| POLD1 | -1,696013231 | 0,003525413 | 0,057065785 |
| SEPT7 | 1,560206331 | 0,003530236 | 0,057065785 |
| CHKA | -7,34224508 | 0,003553541 | 0,057287681 |
| CRYBG3 | 1,617320311 | 0,003551972 | 0,057287681 |
| HDLBP | -1,271594865 | 0,003565832 | 0,05737169 |
| TMEM45B | 2,070116308 | 0,003568345 | 0,05737169 |
| EIF2B4 | 1,886984837 | 0,003575108 | 0,057403274 |
| CIC | -1,924602523 | 0,003584006 | 0,057469015 |
| SREBF2 | -1,158915582 | 0,003592239 | 0,05752391 |
| CSNK1G2 | -2,517128275 | 0,00361767 | 0,057545962 |
| FBXO33 | -3,7048188 | 0,003605852 | 0,057545962 |
| LSM3 | 1,67666718 | 0,003617418 | 0,057545962 |
| OPLAH | -3,499864399 | 0,003610468 | 0,057545962 |
| PBDC1 | 1,647782823 | 0,003605328 | 0,057545962 |
| LMNA | -2,103247059 | 0,003658561 | 0,058042049 |
| PRR11 | 2,929551517 | 0,003657682 | 0,058042049 |
| NOC3L | 2,277972526 | 0,003670376 | 0,058152366 |
| RAB13 | 3,073470511 | 0,003714604 | 0,058775256 |
| HSD3B1 | 3,80887888 | 0,003732972 | 0,058987859 |
| FAM50A | 1,711067936 | 0,003747746 | 0,059143191 |
| CDON | 5,248201085 | 0,003754136 | 0,059165981 |
| CLSPN | 1,337936413 | 0,003762513 | 0,059219977 |
| KIAA2013 | -1,740936815 | 0,00376756 | 0,059221486 |
| NAP1L1 | 1,075536775 | 0,003777163 | 0,059294513 |
| DSEL | -7,313525756 | 0,00380485 | 0,059572794 |
| MAZ | -2,851668483 | 0,003818825 | 0,059713444 |
| CARMIL1 | -1,562553789 | 0,003829515 | 0,059802425 |
| AKAP12 | 0,856153031 | 0,003836636 | 0,059835508 |
| CD58 | 1,830419308 | 0,003859436 | 0,060112729 |
| TDRD9 | -3,285966381 | 0,003885896 | 0,060446141 |
| ENY2 | 1,961503923 | 0,003907425 | 0,060702108 |
| AIMP1 | 1,525458829 | 0,00395091 | 0,061298039 |
| C14H8orf34 | 3,075004192 | 0,003969167 | 0,061501518 |
| BCL2L11 | -4,073478404 | 0,003982425 | 0,061627124 |
| PDIA4 | -2,000419728 | 0,004011622 | 0,061998742 |
| PCBP4 | -5,554343241 | 0,004046759 | 0,062461074 |
| FLT3LG | 3,907113161 | 0,004053215 | 0,062480104 |
| ACVR1B | -7,305525522 | 0,004103884 | 0,063107328 |
| MCC | -3,537395853 | 0,004109191 | 0,063107328 |
| TET3 | -2,235595941 | 0,004109732 | 0,063107328 |
| H2AFY | 2,422478712 | 0,004128476 | 0,063250616 |
| RTTN | -2,642911018 | 0,004129638 | 0,063250616 |
| EIF2AK1 | 1,471643359 | 0,00415639 | 0,063578955 |
| NDUFA4 | 1,171055729 | 0,004170941 | 0,06372005 |
| TBC1D24 | -7,269290179 | 0,004176816 | 0,063728417 |
| POLE | -2,239874153 | 0,00418413 | 0,063758684 |
| KIF24 | 1,947474285 | 0,004251819 | 0,064707715 |
| HYPK | 1,792661527 | 0,004282691 | 0,065094722 |
| DYNC1H1 | -0,99841467 | 0,004304667 | 0,065345719 |
| ATP5PB | 1,099233564 | 0,004350558 | 0,065875154 |
| WDSUB1 | -7,261685116 | 0,004345865 | 0,065875154 |
| DNAJC21 | 1,473618791 | 0,004372037 | 0,066116701 |
| SLC8B1 | -4,910339967 | 0,004410496 | 0,066614087 |
| CRYBG2 | -7,256139714 | 0,004436229 | 0,066918256 |
| SMC6 | 1,302903461 | 0,004445025 | 0,06696649 |
| DDX5 | 1,002483752 | 0,004469326 | 0,067247895 |
| DNAJA2 | 1,241765909 | 0,004484604 | 0,067308448 |
| TNRC18 | -1,456644491 | 0,004479999 | 0,067308448 |
| TEPSIN | -7,250561384 | 0,004509851 | 0,067602546 |
| TNFSF15 | 4,014225722 | 0,00451896 | 0,067654314 |
| LRRK1 | -7,251738722 | 0,004561777 | 0,067973662 |
| PDS5B | 1,360675356 | 0,004561378 | 0,067973662 |
| SLC6A20 | -1,383233823 | 0,004561325 | 0,067973662 |
| TBC1D2B | -7,238517816 | 0,00456302 | 0,067973662 |
| MED1 | 1,120827905 | 0,004584007 | 0,068201363 |
| CNRIP1 | 3,596576935 | 0,004612299 | 0,068265911 |
| GNB2 | -1,747749208 | 0,00461688 | 0,068265911 |
| LPAR2 | -2,223914434 | 0,004594089 | 0,068265911 |
| SLC25A22 | -3,024540237 | 0,00461484 | 0,068265911 |
| TMEM35B | 3,346289246 | 0,004607528 | 0,068265911 |
| IQSEC1 | -3,42662148 | 0,004627658 | 0,068340801 |
| MFHAS1 | -2,525315892 | 0,004647055 | 0,068438205 |
| SUCO | 1,567617905 | 0,004649883 | 0,068438205 |
| TGM2 | -1,561000731 | 0,004651418 | 0,068438205 |
| gene:ENSBTAG00000052141 | 2,764557597 | 0,004677006 | 0,068645823 |
| NSD2 | -2,13230263 | 0,004675348 | 0,068645823 |
| gene:ENSBTAG00000001143 | 6,942937549 | 0,004712337 | 0,068995068 |
| NELFB | -4,07637379 | 0,004709074 | 0,068995068 |
| SETD1B | -1,733388823 | 0,004755789 | 0,069546154 |
| CCDC34 | 1,752875478 | 0,004792004 | 0,069709029 |
| EHMT2 | -3,08079714 | 0,004785465 | 0,069709029 |
| FOXK1 | -4,895566107 | 0,004796065 | 0,069709029 |
| LARP7 | 1,347219481 | 0,004778568 | 0,069709029 |
| MIER1 | 1,732572583 | 0,004791662 | 0,069709029 |
| GCC2 | 1,34919175 | 0,004816378 | 0,069839251 |
| ZNF592 | -2,319294942 | 0,004816701 | 0,069839251 |
| GDE1 | -1,184798333 | 0,00482919 | 0,069935553 |
| SRC | -2,53922354 | 0,004835466 | 0,069941768 |

**Supplementary table 3 –** Differentially expressed genes (DEGs) of CO vs IA comparison.

| **GeneID** | **logFC** | **PValue** | **FDR** |
| --- | --- | --- | --- |
| C21H15orf39 | -4.49852136334053 | 1.27180485586244e-07 | 0.000180397971378057 |
| CRIP1 | -2.48022683422601 | 1.33362989463353e-07 | 0.000180397971378057 |
| TSC22D1_2 | -3.80879251269123 | 1.36412514065074e-07 | 0.000180397971378057 |
| gene:ENSBTAG00000045939 | -5.7150529336572 | 1.58805095241902e-07 | 0.000189009824356912 |
| FASN | -2.06826933007436 | 1.76936237296549e-07 | 0.000191445008754866 |
| NACC1 | -3.71830042842576 | 2.35038142083017e-07 | 0.000233118663922673 |
| RCOR2 | -9.3625752567623 | 2.68744297912092e-07 | 0.000246045741057671 |
| ATP5F1D | -3.57217499850403 | 2.91562823003415e-07 | 0.000247870051384761 |
| ARHGDIA | -2.94473021621171 | 3.45667849376784e-07 | 0.000266148855033567 |
| CSAD | -9.32752930753374 | 3.57787067764835e-07 | 0.000266148855033567 |
| DDX41 | -5.52647573876677 | 5.61177871009993e-07 | 0.000392890530632996 |
| TPT1 | 1.54144386513946 | 6.00205762294026e-07 | 0.000396869387934639 |
| RPS17 | 1.60825677563283 | 6.74155413996894e-07 | 0.00041087664202004 |
| HSPG2 | -4.49224726868967 | 6.90432939035524e-07 | 0.00041087664202004 |
| RPS20 | 1.63141168156811 | 1.09972685060925e-06 | 0.00062328328456911 |
| DEK | 1.99863365756395 | 1.38542353907094e-06 | 0.000749514134637376 |
| ATN1 | -2.1963902607818 | 2.02480714161677e-06 | 0.00104779367824012 |
| gene:ENSBTAG00000023792 | 4.2615639475361 | 2.22136498239601e-06 | 0.00106301886128113 |
| PRPF38B | 2.05038828222162 | 2.23285763166093e-06 | 0.00106301886128113 |
| FOXP4 | -2.39379816563755 | 3.11004233957631e-06 | 0.00142368168944759 |
| EDF1 | -4.73263232964863 | 3.24501994676798e-06 | 0.00143045286690491 |
| CD3EAP | 3.16229684396677 | 3.39699398214862e-06 | 0.00144396508484046 |
| TBL3 | -5.0433041063724 | 3.53509220849059e-06 | 0.00145085060225707 |
| MYL6 | 1.86403658483018 | 3.82566144413659e-06 | 0.00148526560912207 |
| EIF3A | 1.43680741579392 | 3.86852914491549e-06 | 0.00148526560912207 |
| RPL36A | 1.52544782113597 | 4.0880199598016e-06 | 0.00152048792379871 |
| MCM2 | -2.80577595026503 | 6.81220880299736e-06 | 0.00244910833831556 |
| SREK1 | 2.08849507235866 | 6.99627655038893e-06 | 0.00244910833831556 |
| SMC2 | 1.63182653260834 | 7.33510981593275e-06 | 0.00245125134922681 |
| RPS25 | 1.34316292365583 | 7.53003654192254e-06 | 0.00245125134922681 |
| HSPA4 | 1.63307842882557 | 7.62025709304251e-06 | 0.00245125134922681 |
| ZFP36 | -2.3162598223132 | 8.25357429807414e-06 | 0.0025006241596223 |
| PPP4C | -3.12839737808114 | 8.48450981840982e-06 | 0.0025006241596223 |
| UNK | -8.81231913580548 | 8.52022202379049e-06 | 0.0025006241596223 |
| RPS24 | 1.33221047636313 | 8.69513197580863e-06 | 0.0025006241596223 |
| SH3RF1 | -6.91528998694609 | 9.06919659528213e-06 | 0.0025006241596223 |
| SLC6A8 | -2.67501786521473 | 9.25038700068822e-06 | 0.0025006241596223 |
| HSP90B1 | 1.47550668896345 | 9.36825066464788e-06 | 0.0025006241596223 |
| RPL21 | 1.42138997471779 | 9.45455277961717e-06 | 0.0025006241596223 |
| TUBB | -1.54670528747694 | 1.17636632275436e-05 | 0.00301375052273147 |
| RPL22 | 1.5084561266568 | 1.19546881959226e-05 | 0.00301375052273147 |
| PRRC2A | -1.98518516913005 | 1.21542618964132e-05 | 0.00301375052273147 |
| PPIG | 2.0155857713809 | 1.31675270709539e-05 | 0.00319836545303048 |
| TBC1D31 | 2.66310694732038 | 1.37311545743875e-05 | 0.00326856403488721 |
| HSPE1 | 1.71275797784783 | 1.41884293380921e-05 | 0.00331118992121513 |
| HIST1H1E_2 | -3.63934170922753 | 1.55178383823882e-05 | 0.00350470524778172 |
| RAD50 | 1.60152843728614 | 1.56065684870132e-05 | 0.00350470524778172 |
| SRSF11 | 1.53283249466605 | 1.69816628071915e-05 | 0.0036824693913971 |
| WIPF2 | -3.35766478027392 | 1.70169565221678e-05 | 0.0036824693913971 |
| TAGLN2 | -2.203913578434 | 1.79337078155093e-05 | 0.00381155340036056 |
| FAM222B | -3.43776422203615 | 1.93300454719909e-05 | 0.00403624914399361 |
| PEA15 | -8.70165247918876 | 2.11105124078143e-05 | 0.00433202273582423 |
| APOA1 | -2.59159458640816 | 2.22562429294896e-05 | 0.00445408890435308 |
| CDC34 | -6.66072092156413 | 2.24538173635679e-05 | 0.00445408890435308 |
| SHANK2 | -3.42177641304232 | 2.3662168304334e-05 | 0.00461683815013415 |
| SEC61G | 1.99199685926096 | 2.47602268073531e-05 | 0.00474143195710568 |
| NISCH | -5.00649245371323 | 2.50974805324868e-05 | 0.00474143195710568 |
| JUP | -2.1959077408588 | 2.55904877661576e-05 | 0.00475903102176261 |
| VAT1 | -2.23391180783322 | 2.62072060275077e-05 | 0.00479874101752918 |
| ATG2A | -3.9001336417053 | 2.70286197959108e-05 | 0.00487416110319592 |
| RALY | -2.40675168386719 | 2.99383136581166e-05 | 0.00531829565908812 |
| ERCC2 | -3.28321376962002 | 3.06374304851203e-05 | 0.00536245143579267 |
| NCL | 1.22733476903598 | 3.46754892816922e-05 | 0.00598127062943045 |
| gene:ENSBTAG00000008858 | -3.08954250667293 | 3.56105226911948e-05 | 0.00605480630100858 |
| IK | 1.84475990026704 | 3.64851007525964e-05 | 0.00611613618531552 |
| ZBTB40 | -8.53014105648456 | 4.09960140116736e-05 | 0.00677686887176304 |
| ZNF335 | -4.84433115162689 | 4.32886656930708e-05 | 0.00700730347453075 |
| ATP5MC1 | -2.43496105179789 | 4.42201071892955e-05 | 0.00700730347453075 |
| TAF10 | 2.48169869845388 | 4.48060118739631e-05 | 0.00700730347453075 |
| gene:ENSBTAG00000048743 | -8.51069515728987 | 4.53299722590297e-05 | 0.00700730347453075 |
| ATRX | 1.4868684317384 | 4.57203993487419e-05 | 0.00700730347453075 |
| CCDC34 | 2.85020006346808 | 4.59225063866072e-05 | 0.00700730347453075 |
| PSIP1 | 1.78233552883105 | 4.68167339246096e-05 | 0.00702717334588989 |
| PPP2R1A | -2.34830167975143 | 4.8763379604231e-05 | 0.00702717334588989 |
| MYADM | -1.47667741439545 | 4.99183785600624e-05 | 0.00702717334588989 |
| gene:ENSBTAG00000046193 | 5.16894790719176 | 5.02839451509459e-05 | 0.00702717334588989 |
| ZNHIT6 | 2.02708953605609 | 5.04640460151561e-05 | 0.00702717334588989 |
| CLPTM1L | -3.25675572802934 | 5.05047787503537e-05 | 0.00702717334588989 |
| ACTN4 | -1.62115129731015 | 5.06849216468342e-05 | 0.00702717334588989 |
| CCN1 | 3.03844672246339 | 5.08133629000711e-05 | 0.00702717334588989 |
| SOS 1 | 1.9259937607062 | 5.16188835570725e-05 | 0.00702717334588989 |
| PRPF40A | 1.59052904760672 | 5.19569193781138e-05 | 0.00702717334588989 |
| PNN | 1.40902972122787 | 5.29975963312388e-05 | 0.00708738642173487 |
| gene:ENSBTAG00000022275 | 3.5031969595533 | 5.71105351194044e-05 | 0.00755255098879056 |
| RPL24 | 1.21947179062369 | 6.54742851468716e-05 | 0.00849659379025148 |
| RPL23 | 1.14883900733817 | 6.56769138550778e-05 | 0.00849659379025148 |
| LAMA5 | -3.6303980075223 | 6.81281940794198e-05 | 0.00871894371971241 |
| NR2C2 | -4.16416005687902 | 7.23493456882349e-05 | 0.00900162752231373 |
| PPT1 | 1.91881261376429 | 7.27412004479036e-05 | 0.00900162752231373 |
| RPS27 | 1.20880124660461 | 7.29626901590625e-05 | 0.00900162752231373 |
| PTBP1 | -1.33183327320974 | 7.33622811010277e-05 | 0.00900162752231373 |
| ZNF618 | 7.093238303907 | 7.6664366286704e-05 | 0.00927512030663605 |
| EXOSC6 | -5.08101234324841 | 7.71497992234052e-05 | 0.00927512030663605 |
| WDR87 | 4.7614139464334 | 7.8335283325354e-05 | 0.00932346542138363 |
| EEA1 | 1.53479939665342 | 8.08159911572979e-05 | 0.00943077188853816 |
| AIF1L | -2.89401410719456 | 8.08216041531585e-05 | 0.00943077188853816 |
| ROCK1 | 1.70762314031211 | 8.61764344572291e-05 | 0.00988382727485271 |
| KLF9 | -8.53305377702289 | 8.63651517883282e-05 | 0.00988382727485271 |
| RPL5 | 1.21506994137369 | 8.79357530046109e-05 | 0.00989096267053289 |
| BCL2L1 | -3.13222347318914 | 8.80895683982933e-05 | 0.00989096267053289 |
| NOP58 | 1.39658321630074 | 9.25505657369681e-05 | 0.0102638781630794 |
| SLTM | 1.65332467352901 | 9.38181608301177e-05 | 0.0102638781630794 |
| SH3GL1 | -2.06926079462585 | 9.46184846046837e-05 | 0.0102638781630794 |
| HSP90AA1 | 1.42204675061177 | 9.54444110287284e-05 | 0.0102638781630794 |
| MYL9 | -1.86232255570389 | 9.63419367981683e-05 | 0.0102638781630794 |
| RPS6 | 1.12046670488148 | 9.65849734720969e-05 | 0.0102638781630794 |
| FIP1L1 | 1.66295903963283 | 9.99097708109997e-05 | 0.010491664045454 |
| AKT2 | -2.87860767542706 | 0.000100491488924698 | 0.010491664045454 |
| UBE2S | -3.55013442588333 | 0.000102601174340576 | 0.0105643940464487 |
| ROCK2 | 1.40338848720771 | 0.000102963343084192 | 0.0105643940464487 |
| TRMT61A | -5.74896886963238 | 0.000105521720223903 | 0.0107343548214094 |
| TLE3 | -2.32380113166975 | 0.000108195046011534 | 0.0109130291324515 |
| CCDC186 | 1.47177064151392 | 0.000110888276667206 | 0.0110848281616828 |
| gene:ENSBTAG00000050274 | 1.3483357080628 | 0.000111760996420932 | 0.0110848281616828 |
| CD55 | 1.89310776709571 | 0.000113722355879987 | 0.0111164179511622 |
| TNKS1BP1 | -4.98562870400265 | 0.000114582746968365 | 0.0111164179511622 |
| EIF5B | 1.41281388103589 | 0.000114881482775412 | 0.0111164179511622 |
| EIF3M | 1.83008004961329 | 0.000121676204285498 | 0.0116789530919838 |
| PHC3 | 1.94435096546355 | 0.000122724868074633 | 0.0116853710385943 |
| SRM | -1.81224791908993 | 0.000125370284108341 | 0.0118425168369641 |
| PHGDH | 2.96147415139189 | 0.000130298990574701 | 0.0121699383828017 |
| WAC | 1.35810461822766 | 0.00013088154200963 | 0.0121699383828017 |
| BOP 1 | -5.34713009011047 | 0.00014029785776602 | 0.0128910573076641 |
| CAMSAP3 | -2.3861920025662 | 0.000140803012098499 | 0.0128910573076641 |
| PEX19 | 1.79558744989816 | 0.000145915711175006 | 0.0132427187141514 |
| TOP2A | 1.30797644487864 | 0.00014686933878911 | 0.0132427187141514 |
| CD63 | -3.13118223852518 | 0.000151102066338509 | 0.0134953720722907 |
| DNAJC21 | 2.01746501395932 | 0.000151939157930344 | 0.0134953720722907 |
| PMM2 | -3.26899444177909 | 0.000153291528724041 | 0.0135041381224706 |
| SLK | 1.50542636814621 | 0.000154307073152076 | 0.0135041381224706 |
| RPL34 | 1.22932540908629 | 0.000156400375174338 | 0.0135559229970575 |
| DENND6A | 3.17345907596605 | 0.000157699233768498 | 0.0135559229970575 |
| GOLGB1 | 1.20091826253206 | 0.00015831568615283 | 0.0135559229970575 |
| CNTNAP1 | -3.29919833895417 | 0.000160048676981437 | 0.0136064239530933 |
| RPL13A | 1.14238147433741 | 0.000164367099900621 | 0.0136075071646301 |
| PRKCSH | -3.19186400210598 | 0.000164616793756252 | 0.0136075071646301 |
| ASB8 | 2.87451795949243 | 0.000164744523369694 | 0.0136075071646301 |
| SALL4 | -2.16837452804556 | 0.000165556614805744 | 0.0136075071646301 |
| MFAP1 | 2.0102786352109 | 0.000165777897737469 | 0.0136075071646301 |
| PIGQ | -4.18756716068833 | 0.000172098199044406 | 0.0140228156491119 |
| SYT17 | 3.73084553000051 | 0.000173401671873378 | 0.0140228156491119 |
| ADGRL1 | -8.27144889448596 | 0.000174372098476606 | 0.0140228156491119 |
| TRIP11 | 1.71163802885587 | 0.000181064781218829 | 0.0144451612757358 |
| RAB11FIP1 | -1.8523324977662 | 0.00018205126796844 | 0.0144451612757358 |
| CACYBP | 1.75205049155079 | 0.000195260472509285 | 0.01539066320401 |
| RPS3A | 1.1524354101367 | 0.000200226259730758 | 0.0156782430481282 |
| RAB5B | -2.02245097786151 | 0.000203752524260201 | 0.0158500819852609 |
| KIRREL1 | -2.38987945125535 | 0.000206178405122528 | 0.0159346453101839 |
| CHKA | -8.20035789196343 | 0.000212092226413308 | 0.0162415165747386 |
| HIST1H2AC | -2.67474412179084 | 0.000212878220942633 | 0.0162415165747386 |
| YIPF2 | -4.21253473299387 | 0.000214361786769827 | 0.0162505349435318 |
| DNAJA1 | 1.37291075541614 | 0.000217133100578857 | 0.016356444070187 |
| gene:ENSBTAG00000048800 | -6.3340917049246 | 0.00023000302921048 | 0.0170418603680522 |
| CENPF | 1.23120179872201 | 0.00023099396741466 | 0.0170418603680522 |
| PUS7L | 4.81279040469812 | 0.000231504230066704 | 0.0170418603680522 |
| AREL1 | -8.21399227033646 | 0.00023306126990115 | 0.0170418603680522 |
| ZMYND19 | -4.56407092092162 | 0.000233391298940725 | 0.0170418603680522 |
| CACNA1H | -3.6010995863316 | 0.00023630736638912 | 0.0171059768841753 |
| TMSB4X | 1.28508304178067 | 0.000239471166532755 | 0.0171059768841753 |
| SHC1 | -2.47216757105317 | 0.000239853398110296 | 0.0171059768841753 |
| RPL35 | 1.13501546382103 | 0.000240018327983303 | 0.0171059768841753 |
| KMT2D | -1.5236973981051 | 0.000243530326717558 | 0.0172529639797165 |
| RSL1D1 | 1.36053601177556 | 0.000251019610752106 | 0.0175921606455491 |
| NSRP1 | 1.91392251152794 | 0.000251274349667564 | 0.0175921606455491 |
| RBBP6 | 1.40065301678759 | 0.00025394361985587 | 0.0176001213320962 |
| MNT | -4.31967070834086 | 0.00025434556117632 | 0.0176001213320962 |
| gene:ENSBTAG00000048931 | 1.95489633923944 | 0.000265739902356494 | 0.0182822908546069 |
| ACVR2B | -3.68946140175386 | 0.000271243674486406 | 0.0185536908835472 |
| POLE | -2.6394411773124 | 0.00028413169983843 | 0.0193242028084399 |
| RPL29 | -1.46728461798437 | 0.000297030238235672 | 0.0200866698606873 |
| AMOT | -5.4745099611043 | 0.00030535900610067 | 0.020533236670114 |
| HSCB | 3.03443501598303 | 0.0003196217650647 | 0.021264555818223 |
| PELP1 | -4.38904127431336 | 0.000319808056751967 | 0.021264555818223 |
| GRAMD1A | -2.56315273145473 | 0.000330887515484389 | 0.0218790178294178 |
| NAV2 | -2.03630166299224 | 0.000343587932368057 | 0.0225251584531182 |
| SHF | -3.38360341019739 | 0.000344444533563057 | 0.0225251584531182 |
| SLC27A4 | -3.29298797917286 | 0.000360053532780507 | 0.0232364240875922 |
| SETD1A | -2.1153650862975 | 0.000360177101785499 | 0.0232364240875922 |
| LUC7L3 | 1.44909374989251 | 0.000361177823576253 | 0.0232364240875922 |
| CSPP1 | 1.69793572821841 | 0.000364822678217786 | 0.0233447285814413 |
| RPS7 | 1.03967398156941 | 0.000371017395952082 | 0.0234926379669852 |
| NAPA | -4.34791485690616 | 0.000371081829759135 | 0.0234926379669852 |
| AGO2 | -2.93686712995808 | 0.000374310425021723 | 0.0235716543841722 |
| CLDN4 | -1.89618528107242 | 0.00037713315387131 | 0.0236244147230333 |
| GNL3 | 1.52531765484986 | 0.000384248893840822 | 0.0239441378769291 |
| PRRC2C | 0.929860902129171 | 0.000391871619622599 | 0.0241401495987491 |
| CYHR1 | -5.53139412064362 | 0.000393209343513781 | 0.0241401495987491 |
| DNTTIP2 | 1.43717665094126 | 0.000395312432573265 | 0.0241401495987491 |
| gene:ENSBTAG00000024999 | -2.5065376885602 | 0.000395507408146201 | 0.0241401495987491 |
| COX6B1 | -1.88759650320051 | 0.000402452904543702 | 0.0244387472953018 |
| ETHE1 | -2.12189245430257 | 0.000413292677876251 | 0.0249695911273256 |
| MTDH | 1.33976866678688 | 0.000423137130858548 | 0.0254352430882749 |
| ERF | -2.34855633783102 | 0.000434115152338566 | 0.0257921507006309 |
| TRAF3 | -8.08111157305554 | 0.000435552598158443 | 0.0257921507006309 |
| FMNL2 | 2.88540765075349 | 0.000435575725997884 | 0.0257921507006309 |
| MAB21L3 | -2.65851644189069 | 0.00044051152495242 | 0.0259552879702163 |
| GATAD2A | -1.60004236916329 | 0.000451623347845233 | 0.0263555491527864 |
| JAK1 | 1.671247177827 | 0.000452463290287743 | 0.0263555491527864 |
| SAMD4B | -2.24975249895085 | 0.00045394787231736 | 0.0263555491527864 |
| HGH1 | -2.32664551289271 | 0.000469138951367619 | 0.0271052999960068 |
| KANSL3 | -3.49203494296481 | 0.000486557259042748 | 0.0279758671358782 |
| PCID2 | -5.37012107230426 | 0.000498733096796536 | 0.028483165970603 |
| UBN2 | 1.45656064544647 | 0.000501714596245636 | 0.028483165970603 |
| ACSS3 | 8.07252130860989 | 0.000504968616191729 | 0.028483165970603 |
| gene:ENSBTAG00000052021 | 8.38721643212167 | 0.00050518102781569 | 0.028483165970603 |
| RPP30 | 2.04247136208566 | 0.000509639302530997 | 0.028483165970603 |
| PDP2 | -2.28225948373925 | 0.0005104320692114 | 0.028483165970603 |
| SF3B4 | -1.70202108941996 | 0.00051357618613263 | 0.028483165970603 |
| HABP4 | 2.37107121669925 | 0.000514525347309667 | 0.028483165970603 |
| GOLGA4 | 1.06125067741979 | 0.000524996781686674 | 0.0289282948871981 |
| ZFAND5 | 1.19314170075573 | 0.000530001269967224 | 0.0290694705767277 |
| CORO1B | -8.1531570635671 | 0.000544150873868743 | 0.0295271800114479 |
| BCL9L | -4.05331425199156 | 0.000544291375166047 | 0.0295271800114479 |
| SMC5 | 1.79144311555431 | 0.00054757008502995 | 0.0295271800114479 |
| DOT1L | -2.58012340243266 | 0.000548269768318768 | 0.0295271800114479 |
| gene:ENSBTAG00000003367 | -2.7050279806706 | 0.000554924378375755 | 0.0297509457271542 |
| ABTB2 | -4.44554342796008 | 0.000564050473770197 | 0.0301046131785331 |
| PRKN | -7.96672779217653 | 0.000591558268587565 | 0.0313686331515874 |
| UBR4 | -1.25936507509702 | 0.000593004743665533 | 0.0313686331515874 |
| FTL | -1.0904506421839 | 0.000596254865858843 | 0.0314009974046547 |
| CETN2 | 2.37809197499105 | 0.000613866034644168 | 0.0321860508561008 |
| RPL27 | 1.12841638824553 | 0.000620869890163486 | 0.0324104975119553 |
| JUNB | -2.72665398590693 | 0.000631112387249636 | 0.0325997859697125 |
| HCN2 | -3.81358233801317 | 0.000631971081764407 | 0.0325997859697125 |
| PRRC2B | -1.9655527842638 | 0.00063391436677204 | 0.0325997859697125 |
| UACA | 1.3386133474348 | 0.000637232268076309 | 0.0325997859697125 |
| ATG7 | -4.74982324600827 | 0.000638191071327761 | 0.0325997859697125 |
| RPTOR | -2.61046438214129 | 0.000644792749343572 | 0.0327962534302871 |
| CYCS | 1.42420207119298 | 0.000651489941371587 | 0.0328643225191086 |
| PLEC | -2.20088638113081 | 0.000651653513233879 | 0.0328643225191086 |
| PCBP1 | -1.70840249189737 | 0.000662843752868742 | 0.0332876217157965 |
| RALGAPB | 1.69267348721028 | 0.000671650301549715 | 0.0335881591976668 |
| GTF3C3 | 2.41817205502444 | 0.000674859230951628 | 0.0336074249656329 |
| ARHGEF7 | -3.92012713020474 | 0.000696644750286986 | 0.0345477742413155 |
| TANGO6 | -6.03383965639241 | 0.000703459545743541 | 0.0347409772341893 |
| CD2AP | 1.18546937438677 | 0.000709297301995552 | 0.0348845309435994 |
| HERC2 | -1.68916630133626 | 0.000720910137783656 | 0.0353097632094695 |
| G3BP1 | -1.83185645483903 | 0.00072829017302037 | 0.0355250395052805 |
| REXO1 | -3.18287083267268 | 0.000733270924565738 | 0.0356220022211486 |
| TMX3 | -4.70823754791622 | 0.000741845568556462 | 0.0357785911285273 |
| SUV39H1 | -6.20399286413188 | 0.000743897523984383 | 0.0357785911285273 |
| PTPN23 | -3.03434292357276 | 0.000748288937521215 | 0.0357785911285273 |
| PLEKHA7 | -2.80225783529521 | 0.000748518668375339 | 0.0357785911285273 |
| RPL7 | 0.966391719525827 | 0.000752629345922646 | 0.0357858516121398 |
| RAF1 | -2.33420578895136 | 0.000754683982074196 | 0.0357858516121398 |
| BEND3 | -2.77839486253372 | 0.000762270864292677 | 0.0360021739159184 |
| NELFB | -4.520578889497 | 0.000773939288044854 | 0.0364087960723709 |
| RPS21 | 1.13843478030344 | 0.000782012250041382 | 0.0366271470598427 |
| NAA11 | -7.87778937029335 | 0.000787903913545554 | 0.0366271470598427 |
| SALL1 | -3.56082549614141 | 0.000788969578312252 | 0.0366271470598427 |
| SCAP | -2.42410412128393 | 0.000790890337286135 | 0.0366271470598427 |
| MEF2D | -2.53402157235549 | 0.000796276822124888 | 0.0366964858227828 |
| PSMC2 | 1.5802140799567 | 0.000798554010090804 | 0.0366964858227828 |
| RAN | 1.18117225756349 | 0.000803829153766131 | 0.0367968253389404 |
| SKA3 | 1.7844636787795 | 0.000809816782042644 | 0.0369288863596611 |
| ALG3 | -4.37431139466399 | 0.00081715839392595 | 0.0370974655807404 |
| CCHCR1 | 2.40129987983087 | 0.000819747391004429 | 0.0370974655807404 |
| SLC12A4 | -2.77567596464911 | 0.000823498381439791 | 0.0371260520299106 |
| gene:ENSBTAG00000054942 | 1.66843489908407 | 0.000833496411356443 | 0.0374349973130732 |
| FRS2 | 2.09141807650034 | 0.000843420810124921 | 0.0375285458137728 |
| MFSD6L | 6.22928626257216 | 0.000845758214828451 | 0.0375285458137728 |
| ZNF638 | 1.26751393662227 | 0.000846899933100189 | 0.0375285458137728 |
| SLC4A11 | -4.07866861128841 | 0.000848191801706006 | 0.0375285458137728 |
| FNBP1L | 1.39146039937091 | 0.000851814896932494 | 0.0375492626047798 |
| TP53 | -1.92104834974244 | 0.000869321045929309 | 0.0381795538326592 |
| SND1 | -1.29187632502248 | 0.000885965142236183 | 0.0386781760718398 |
| COL4A2 | -2.63876228369345 | 0.000891585770248454 | 0.0386781760718398 |
| PLK2 | 1.22635623861952 | 0.000894288833863056 | 0.0386781760718398 |
| BICRAL | -2.07415962033315 | 0.000898101103658835 | 0.0386781760718398 |
| RPS15A | 1.10783046914447 | 0.000899634714412177 | 0.0386781760718398 |
| DUSP14 | 2.00765661483293 | 0.000909317024898744 | 0.0386781760718398 |
| MED6 | 2.3307782370122 | 0.000911004400590944 | 0.0386781760718398 |
| EIF1AX | 1.84657579856852 | 0.000911130933002184 | 0.0386781760718398 |
| LONP1 | -1.89500533804616 | 0.000913021168424728 | 0.0386781760718398 |
| GGTA1 | 1.52256616236221 | 0.000913171523793226 | 0.0386781760718398 |
| FLNB | -0.945564695349072 | 0.000924690789322435 | 0.0390271977819703 |
| MAP3K3 | -1.95866759590885 | 0.000960727173391675 | 0.0402962901267953 |
| MYO19 | -2.00436022526576 | 0.000961531372543258 | 0.0402962901267953 |
| RNF40 | -3.79951711136678 | 0.000971805443336351 | 0.0404559246048979 |
| IL2RB | -3.19412007965208 | 0.000973860797746309 | 0.0404559246048979 |
| SRRD | -7.78447829090649 | 0.00097553775513407 | 0.0404559246048979 |
| SMG5 | -1.97845401924882 | 0.000979672328208654 | 0.040486319619234 |
| PHOSPHO1 | -3.19255760235289 | 0.00101821577098711 | 0.0419335782224519 |
| RPL6 | 0.90963267725555 | 0.00102667813490903 | 0.0421362867644388 |
| STX2 | 3.15382148762006 | 0.00103455688646455 | 0.0421677345979449 |
| ZC3H10 | 1.80580068792305 | 0.00103665222375762 | 0.0421677345979449 |
| TRRAP | -1.43449909261451 | 0.00104151195642415 | 0.0421677345979449 |
| RBBP8 | 1.612668025004 | 0.00104266856697632 | 0.0421677345979449 |
| SLC7A4 | -1.91506235557311 | 0.00104523393844112 | 0.0421677345979449 |
| ZCCHC17 | 1.56409199279325 | 0.00105449349112457 | 0.0421677345979449 |
| UBIAD1 | -3.36705485721816 | 0.00105588855361405 | 0.0421677345979449 |
| FLT3LG | 6.64977166294595 | 0.00105981968845099 | 0.0421677345979449 |
| ATG3 | 1.99339389723295 | 0.00105996929743644 | 0.0421677345979449 |
| TEPSIN | -8.10164670832279 | 0.00106287349851987 | 0.0421677345979449 |
| SNRPA | -2.28437472261626 | 0.00109082056077387 | 0.0431327120077427 |
| PFAS | -2.77762347685921 | 0.00109643684755541 | 0.0432112296675646 |
| WIZ | -3.08003182725809 | 0.00111648683460859 | 0.0437048277454207 |
| PLXNA1 | -7.92204474729238 | 0.00111854231138069 | 0.0437048277454207 |
| NID2 | 2.46815831385052 | 0.00112559376850737 | 0.0437048277454207 |
| gene:ENSBTAG00000015551 | -3.2548116873173 | 0.0011256260218748 | 0.0437048277454207 |
| LRCH4 | -3.6269299593771 | 0.00112732163651858 | 0.0437048277454207 |
| POLR2L | -2.27253198881086 | 0.00114639226158345 | 0.0442214621305502 |
| SEC23A | 2.59709474984702 | 0.00114807862530163 | 0.0442214621305502 |
| NAP1L1 | 1.13280661390868 | 0.00116498237969006 | 0.0447002393572552 |
| CCAR1 | 1.24205108889945 | 0.00116802003361673 | 0.0447002393572552 |
| ATXN2L | -1.34918563988892 | 0.00117287887136774 | 0.0447423215609577 |
| CAPN5 | -2.19022233959424 | 0.00119731539193066 | 0.0455285872037021 |
| C29H11orf24 | -3.42600940910103 | 0.00120841487695629 | 0.0458043116736745 |
| RAP1GDS1 | 2.14288371138975 | 0.00121839514790113 | 0.045872692904321 |
| CCDC112 | 2.7496622974924 | 0.00122023396021371 | 0.045872692904321 |
| RHBDF1 | -2.02109843383827 | 0.00122527395435755 | 0.045872692904321 |
| FABP5 | 5.24936308726164 | 0.00123017483309115 | 0.045872692904321 |
| FOXRED2 | -3.92759035418434 | 0.00123178569444527 | 0.045872692904321 |
| LRRC8D | 1.91453456917722 | 0.00123334412110424 | 0.045872692904321 |
| GLUL | -1.20843393279433 | 0.00124778096085565 | 0.0462650747542178 |
| SAT1 | 2.64141776758043 | 0.00125509435451444 | 0.0463917174143814 |
| VBP1 | 1.75192722299869 | 0.00126236560769219 | 0.0464569628318553 |
| DNAJB6 | -2.62163149454446 | 0.00126793135270684 | 0.0464569628318553 |
| PCNX3 | -1.80416082912136 | 0.00126856939340892 | 0.0464569628318553 |
| RAVER1 | -2.06215187181993 | 0.00128062840016335 | 0.04675472152989 |
| PLXNB2 | -1.44097308418143 | 0.0013064974038434 | 0.0474667419452244 |
| SOWAHB | -5.86013703070493 | 0.00130810715493477 | 0.0474667419452244 |
| WAPL | 1.21352401014551 | 0.00132081765219263 | 0.0477822847914791 |
| CCT4 | 1.13960941104775 | 0.00132656924314734 | 0.0478449307028474 |
| SMC3 | 1.25008421924567 | 0.0013371589064618 | 0.048081164062563 |
| FIS1 | -2.82387350179658 | 0.00134757063380716 | 0.0481796390258424 |
| PHF13 | -7.67813015220158 | 0.00134799359734545 | 0.0481796390258424 |
| LYPLA2 | -2.70814620682546 | 0.00136031856476923 | 0.0484131001899394 |
| HHEX | 6.42869760894216 | 0.00136266077664508 | 0.0484131001899394 |
| CALR | 1.18140343464435 | 0.00137611316811699 | 0.0487455325206202 |
| IGSF9B | -4.53189458469842 | 0.00139618269271549 | 0.0492111313017012 |
| PPCS | -5.88254183909902 | 0.00139752666610444 | 0.0492111313017012 |
| FLNA | -1.63408502845555 | 0.00141495011017895 | 0.049677687939085 |
| ZNF777 | -5.22250146845692 | 0.00142525099319229 | 0.0497189929914255 |
| CDK2 | 2.32450469784641 | 0.00142968421070009 | 0.0497189929914255 |
| PI4K2A | -2.98128855437434 | 0.00143509567854565 | 0.0497189929914255 |
| SMC6 | 1.38638442958742 | 0.00143856352161205 | 0.0497189929914255 |
| NASP | 0.948801365238426 | 0.00144078598652879 | 0.0497189929914255 |
| TMEM222 | -7.64347550491431 | 0.00144119077315088 | 0.0497189929914255 |
| CPSF6 | -1.70607225801557 | 0.00144866015611267 | 0.0498322346186504 |
| TRAPPC6A | -7.68777448431153 | 0.00145718607148406 | 0.0499810623135541 |
| KMT2E | 1.22078916044387 | 0.00147074382586253 | 0.0503011293546433 |
| HSPD1_1 | 0.980474746374732 | 0.00147555348652055 | 0.050321024631999 |
| UBE2V2 | 1.87302200418148 | 0.00148185388952714 | 0.0503914999804344 |
| SELENOK | 2.09513588457136 | 0.00155158862135373 | 0.0526125577531399 |
| TPM4 | 0.939904975913385 | 0.00158156151851044 | 0.0533106712498922 |
| gene:ENSBTAG00000051208 | 2.21500286637166 | 0.00158801775712892 | 0.0533106712498922 |
| BCL2L11 | -4.31839358701342 | 0.00158870444215879 | 0.0533106712498922 |
| ZBTB9 | -3.6915469282991 | 0.00159009311827523 | 0.0533106712498922 |
| HNRNPA1 | 0.98005741340286 | 0.00161630734251351 | 0.0540373314342577 |
| TIMM17B | -2.62316007924924 | 0.00162671646625829 | 0.0540407867366979 |
| ESPL1 | -1.7292820830916 | 0.00163290868647892 | 0.0540407867366979 |
| PURB | -2.36739680999439 | 0.00163334404272158 | 0.0540407867366979 |
| HIST1H2BN | -1.55551243945251 | 0.00163457261176367 | 0.0540407867366979 |
| AKT1 | -5.19054124271562 | 0.00165536812279604 | 0.0545767074723502 |
| RPS16 | 0.893022440496476 | 0.0016797100737718 | 0.0552262687790938 |
| MYO1C | -1.62664422164411 | 0.00168846812405796 | 0.0553612881888093 |
| SASS6 | 1.55512584136414 | 0.0016975497205973 | 0.055506144985025 |
| PRMT1 | -2.36363412935255 | 0.00170872194908988 | 0.0557183798303225 |
| NDUFA5 | 1.89104749129659 | 0.00171983396741773 | 0.055927496940453 |
| ADH5 | 1.55831029781013 | 0.00173910700671465 | 0.0564001405828822 |
| TLK2 | 1.594907063613 | 0.00175408799325291 | 0.0565216762936205 |
| NR1H2 | -2.18639139793539 | 0.00175870605669182 | 0.0565216762936205 |
| AP2A1 | -3.84739392656022 | 0.0017602260020457 | 0.0565216762936205 |
| C7H19orf67 | -2.17087026022484 | 0.00176185026927686 | 0.0565216762936205 |
| RACK1 | 0.895270331462583 | 0.00177940138534725 | 0.0568030690712222 |
| CDK2AP1 | 1.68572509125022 | 0.00178016675882758 | 0.0568030690712222 |
| DBI | 1.21030579910001 | 0.00178515353767286 | 0.0568098861106481 |
| KCTD15 | -3.02349076276859 | 0.00179940975671305 | 0.0571108664650633 |
| RPL26 | 0.994804940086023 | 0.00182032170773795 | 0.0576209280997263 |
| MEF2C | 6.09512749650514 | 0.00184109987015587 | 0.0581121487729556 |
| CCT8 | 1.13706309754028 | 0.00184954985922127 | 0.0581121487729556 |
| DNM2 | -1.80205736196737 | 0.00185048768147792 | 0.0581121487729556 |
| SKI | -2.99341967118148 | 0.00187357568027748 | 0.0586823624912173 |
| SNX4 | 1.68057571962759 | 0.0019029866734059 | 0.0594471060023018 |
| PIN1 | -2.83652208307775 | 0.00197107320189189 | 0.0612589119947556 |
| BAZ1B | 1.03173258249338 | 0.00197127905343567 | 0.0612589119947556 |
| ABCF3 | -4.15059941936207 | 0.00198049970529184 | 0.0613721006114137 |
| HNRNPD | 1.00632404796338 | 0.0019860992615801 | 0.0613721006114137 |
| LYSMD2 | 3.56352215092619 | 0.001990390760881 | 0.0613721006114137 |
| GTF3C5 | -2.59573910066786 | 0.00200443211325705 | 0.0616453514521586 |
| TCF19 | -7.57813817864197 | 0.00202151080855773 | 0.0619132911663453 |
| ATP6V1E1 | 1.18760157145238 | 0.00202786785421574 | 0.0619132911663453 |
| VWA5B2 | -7.67143881707434 | 0.00202875008863004 | 0.0619132911663453 |
| LZTFL1 | 2.77054580397253 | 0.00206316915626133 | 0.0628026580506966 |
| BRF1 | -4.76093251592506 | 0.00208184687227428 | 0.0632095445760421 |
| PCED1B | -1.52111816426355 | 0.00208959675377428 | 0.0632109235741176 |
| CENPE | 1.28264386646504 | 0.00209389684510262 | 0.0632109235741176 |
| PEX14 | -5.04670218180967 | 0.00209782513962161 | 0.0632109235741176 |
| gene:ENSBTAG00000055145 | -2.66397994380691 | 0.00213072006788765 | 0.063883368566512 |
| PHKG2 | -7.51454038091533 | 0.00213087693840575 | 0.063883368566512 |
| ZNF512B | -7.62060833286635 | 0.00214223034891476 | 0.0640623759115162 |
| RNF126 | -2.79456668292628 | 0.00215703438407277 | 0.0643434166396845 |
| ATP13A4 | -7.71799024766468 | 0.00217426601377665 | 0.0646952852399242 |
| MED4 | 2.02416209637738 | 0.00219319072255593 | 0.0650346138031468 |
| gene:ENSBTAG00000038258 | -6.0501616804806 | 0.002196598449745 | 0.0650346138031468 |
| SMARCA5 | 1.00579128935392 | 0.00221616290585743 | 0.0654510444305587 |
| RPL9 | 0.955481551093094 | 0.00222921621008563 | 0.0656735924070277 |
| LAP3 | 1.80514870720392 | 0.00224132065273369 | 0.0658671565650281 |
| YBX2 | -7.48135360880818 | 0.00225681548761679 | 0.0659335525976472 |
| gene:ENSBTAG00000050077 | -8.39165538440541 | 0.0022588454313468 | 0.0659335525976472 |
| STK11 | -7.48835012699718 | 0.00226397450893279 | 0.0659335525976472 |
| TRIM71 | -2.83913282842223 | 0.00226573878444276 | 0.0659335525976472 |
| RNF145 | 2.38532064200345 | 0.00227741868889359 | 0.0660023405144586 |
| RNF144A | -7.54346316434428 | 0.00227919357683099 | 0.0660023405144586 |
| EIF4H | -1.10541308570536 | 0.00231330240779157 | 0.0666702142455074 |
| DSTN | 1.17422964352006 | 0.0023170837462139 | 0.0666702142455074 |
| INO80D | -1.35815308053511 | 0.0023190613928449 | 0.0666702142455074 |
| DYRK1B | -7.55306200715696 | 0.00233863540694189 | 0.0670709364178852 |
| RPS4X | 0.887493601440273 | 0.00236197938002181 | 0.0674671298626569 |
| KIAA0319L | -3.8797457372584 | 0.00236378702341858 | 0.0674671298626569 |
| SKIDA1 | -4.53495672671284 | 0.00237559728149875 | 0.0676420068047803 |
| TRPC4AP | -1.97981542081468 | 0.00238693868542049 | 0.0678027308684359 |
| ASB9 | 5.70465630019184 | 0.00240477446194027 | 0.0681467277286026 |
| SLC25A37 | -3.75880360717001 | 0.00241199643229352 | 0.0681890297794715 |
| ATP5F1C | 1.23671920304673 | 0.00244911339587741 | 0.0690742835017368 |
| ZNF557 | -5.04520952775691 | 0.00247856800166779 | 0.0697397549783689 |
